# Informed Conditioning on Clinical Covariates Increases Power in Case-Control Association Studies

**DOI:** 10.1371/journal.pgen.1003032

**Published:** 2012-11-08

**Authors:** Noah Zaitlen, Sara Lindström, Bogdan Pasaniuc, Marilyn Cornelis, Giulio Genovese, Samuela Pollack, Anne Barton, Heike Bickeböller, Donald W. Bowden, Steve Eyre, Barry I. Freedman, David J. Friedman, John K. Field, Leif Groop, Aage Haugen, Joachim Heinrich, Brian E. Henderson, Pamela J. Hicks, Lynne J. Hocking, Laurence N. Kolonel, Maria Teresa Landi, Carl D. Langefeld, Loic Le Marchand, Michael Meister, Ann W. Morgan, Olaide Y. Raji, Angela Risch, Albert Rosenberger, David Scherf, Sophia Steer, Martin Walshaw, Kevin M. Waters, Anthony G. Wilson, Paul Wordsworth, Shanbeh Zienolddiny, Eric Tchetgen Tchetgen, Christopher Haiman, David J. Hunter, Robert M. Plenge, Jane Worthington, David C. Christiani, Debra A. Schaumberg, Daniel I. Chasman, David Altshuler, Benjamin Voight, Peter Kraft, Nick Patterson, Alkes L. Price

**Affiliations:** 1 Department of Epidemiology, Harvard School of Public Health, Boston, Massachusetts, United States of America; 2 Department of Biostatistics, Harvard School of Public Health, Boston, Massachusetts, United States of America; 3 Broad Institute of Harvard and Massachusetts Institute of Technology, Cambridge, Massachusetts, United States of America; 4 Program in Molecular and Genetic Epidemiology, Harvard School of Public Health, Boston, Massachusetts, United States of America; 5 Channing Laboratory, Department of Medicine, Brigham and Women's Hospital and Harvard Medical School, Boston, Massachusetts, United States of America; 6 Division of Nephrology, Department of Medicine, Beth Israel Deaconess Medical Center and Harvard Medical School, Boston, Massachusetts, United States of America; 7 Arthritis Research UK Epidemiology Unit, Manchester Academic Health Science Centre, The University of Manchester, Manchester, United Kingdom; 8 Department of Genetic Epidemiology, University Medical Centre, University of Göttingen, Göttingen, Germany; 9 Center for Human Genomics, Wake Forest School of Medicine, Winston-Salem, North Carolina, United States of America; 10 Department of Internal Medicine/Section on Nephrology, Wake Forest School of Medicine, Winston-Salem, North Carolina, United States of America; 11 Roy Castle Lung Cancer Research Programme, University of Liverpool, Liverpool, United Kingdom; 12 Department of Clinical Sciences, Diabetes and Endocrinology Research Unit, Scania University Hospital, Lund University, Malmö, Sweden; 13 Section for Toxicology, National Institute of Occupational Health, Oslo, Norway; 14 Institute of Epidemiology, German Research Centre for Environmental Health, Neuherberg, Germany; 15 Department of Preventive Medicine, University of Southern California Keck School of Medicine, Los Angeles, California, United States of America; 16 Department of Biochemistry, Wake Forest University School of Medicine, Winston-Salem, North Carolina, United States of America; 17 Musculoskeletal Research Programme, Division of Applied Medicine, University of Aberdeen, Aberdeen, United Kingdom; 18 Epidemiology Program, Cancer Research Center, University of Hawaii, Honolulu, Hawaii, United States of America; 19 Division of Cancer Epidemiology and Genetics, National Cancer Institute, National Institutes of Health, Bethesda, Maryland, United States of America; 20 Division of Public Health Sciences, Wake Forest University School of Medicine, Winston-Salem, North Carolina, United States of America; 21 Thoraxklinik am Universitätsklinikum, Heidelberg, Germany; 22 Translational Lung Research Centre Heidelberg (TLRC-H), German Center for Lung Research, Heidelberg, Germany; 23 NIHR–Leeds Musculoskeletal Biomedical Research Unit, Leeds, United Kingdom; 24 DKFZ–German Cancer Research Center, Heidelberg, Germany; 25 King's College Hospital National Health Service Foundation Trust, London, United Kingdom; 26 Liverpool Heart and Chest Hospital, Liverpool, United Kingdom; 27 Deptartment of Infection and Immunity, University of Sheffield, Sheffield, United Kingdom; 28 NIHR Oxford Musculoskeletal Biomedical Research Unit, Nuffield Orthopaedic Centre, Oxford, United Kingdom; 29 Division of Rheumatology, Immunology, and Allergy and Division of Genetics, Brigham and Women's Hospital, Boston, Massachusetts, United States of America; 30 Department of Environmental Health, Harvard School of Public Health, Boston, Massachusetts, United States of America; 31 Division of Preventive Medicine, Department of Medicine, Brigham and Women's Hospital, Boston, Harvard Medical School, Massachusetts, United States of America; 32 Schepens Eye Research Institute, Department of Ophthalmology, Harvard Medical School, Boston, Massachusetts, United States of America; 33 Center for Human Genetic Research, Department of Molecular Biology and Diabetes Unit, Massachusetts General Hospital, Boston, Massachusetts, United States of America; 34 Departments of Genetics and Medicine, Harvard Medical School, Boston, Massachusetts, United States of America; The University of Queensland, Australia

## Abstract

Genetic case-control association studies often include data on clinical covariates, such as body mass index (BMI), smoking status, or age, that may modify the underlying genetic risk of case or control samples. For example, in type 2 diabetes, odds ratios for established variants estimated from low–BMI cases are larger than those estimated from high–BMI cases. An unanswered question is how to use this information to maximize statistical power in case-control studies that ascertain individuals on the basis of phenotype (case-control ascertainment) or phenotype and clinical covariates (case-control-covariate ascertainment). While current approaches improve power in studies with random ascertainment, they often lose power under case-control ascertainment and fail to capture available power increases under case-control-covariate ascertainment. We show that an informed conditioning approach, based on the liability threshold model with parameters informed by external epidemiological information, fully accounts for disease prevalence and non-random ascertainment of phenotype as well as covariates and provides a substantial increase in power while maintaining a properly controlled false-positive rate. Our method outperforms standard case-control association tests with or without covariates, tests of gene x covariate interaction, and previously proposed tests for dealing with covariates in ascertained data, with especially large improvements in the case of case-control-covariate ascertainment. We investigate empirical case-control studies of type 2 diabetes, prostate cancer, lung cancer, breast cancer, rheumatoid arthritis, age-related macular degeneration, and end-stage kidney disease over a total of 89,726 samples. In these datasets, informed conditioning outperforms logistic regression for 115 of the 157 known associated variants investigated (P-value = 1**×**10^−9^). The improvement varied across diseases with a 16% median increase in χ^2^ test statistics and a commensurate increase in power. This suggests that applying our method to existing and future association studies of these diseases may identify novel disease loci.

## Author Summary

This work describes a new methodology for analyzing genome-wide case-control association studies of diseases with strong correlations to clinical covariates, such as age in prostate cancer and body mass index in type 2 diabetes. Currently, researchers either ignore these clinical covariates or apply approaches that ignore the disease's prevalence and the study's ascertainment strategy. We take an alternative approach, leveraging external prevalence information from the epidemiological literature and constructing a statistic based on the classic liability threshold model of disease. Our approach not only improves the power of studies that ascertain individuals randomly or based on the disease phenotype, but also improves the power of studies that ascertain individuals based on both the disease phenotype and clinical covariates. We apply our statistic to seven datasets over six different diseases and a variety of clinical covariates. We found that there was a substantial improvement in test statistics relative to current approaches at known associated variants. This suggests that novel loci may be identified by applying our method to existing and future association studies of these diseases.

## Introduction

Genetic risk in case-control studies often varies as a function of body mass index (BMI), age or other clinical covariates. For example, in a recent type 2 diabetes study, 23 of 29 established associated SNPs had higher odds ratios when estimated from low-BMI cases than from high-BMI cases (average odds ratios 1.182 versus 1.128) [1]. Higher genetic risk in early-onset cases has been shown empirically for prostate and breast cancers [2], [3], and has also been hypothesized for other diseases [4], [5]. Covariates such as smoking status may affect genetic risk in several diseases including lung cancer [6], and information on these covariates may alter the expected level of genetic risk carried by a case (or control) sample.

The question of how to optimally incorporate these covariates in case-control association studies is a function of the study design. We divide the set of possible study designs into three classes, random ascertainment (cohort or cross-section designs), case-control ascertainment that ascertains individuals based on phenotype, and case-control-covariate ascertainment that ascertains on both phenotype and clinical covariate (as in age-matched studies). When individuals are randomly ascertained, conditioning on covariates associated with phenotype can increase study power by reducing phenotypic variance [7]. It is well known that conditioning on covariates in ascertained data can result in a dramatic loss in power [8], [9], [10], [11] , and several approaches to address this issue in case-control studies have previously been described [12], [13], [14]. In addition, a paper just published in *Nature Genetics* [15] has made a valuable contribution by highlighting this issue for both genetic covariates and a clinical covariate (gender) in case-control studies, although that paper did not propose a new method to solve this important problem. Matched case-control-covariate ascertainment is commonly used as a means of preventing ascertainment induced power loss by matching the covariate distribution in cases and controls [13], but standard conditioning provides no gain in power in this case [16]. show that another type of case-control-covariate ascertainment, oversampling low-risk (low-BMI) cases and high-risk (high-BMI) controls can increase power with standard association tests, but standard statistical tests may not capture all of the available power increase. As we show below, previous approaches such as logistic or linear regression (Armitage trend test [17]) with or without covariates, marginal or joint tests of gene x covariate interaction [18], [19], comparing early-onset cases to controls [5], [20], analyzing cases only [21], and a semi-parametric approach designed to address case-control ascertainment issues [12], all fail to capture the increase in statistical power that is available when there exists external epidemiological data describing disease prevalence as a function of the covariate. Some of these previous methods lose power under case-control ascertainment, and all fail to capture the available power gain under case-control-covariate ascertainment.

Here, we investigate a new approach to estimating the parameters of the liability threshold (LT) model [22], a classical modeling approach that has recently been used in studies of heritability and risk prediction [23], [24], [25]. Previously, we developed a parameter estimation method for the LT model in the case of genetic covariates (known associated variants) for which samples are randomly ascertained, and showed that it improved power relative to logistic regression with or without conditioning [26]. In this work, we develop a new parameter estimation method for studies with randomly *or non-randomly* ascertained clinical covariates that leverages the epidemiological literature to fit LT parameters. By estimating covariate effect sizes externally from the case-control study data this approach prevents ascertainment-induced power loss, while maintaining the power gain achieved by reducing phenotypic variance. We show by simulation that our approach to fitting liability threshold models and computing case-control association statistics outperforms previously developed approaches. Our method produces a large improvement in power under case-control-covariate ascertainment, a study design that previous methods do not address [12], [13], [17], [26]. Our method also outperforms previous methods under case-control ascertainment, because covariate effect sizes can be estimated more accurately using external epidemiological information. We demonstrate both analytically and empirically that our association statistic produces the correct null distribution.

We apply the method to empirical case-control ascertained and case-control-covariate ascertained studies for seven different diseases: type 2 diabetes, prostate cancer, lung cancer, post-menopausal breast cancer, rheumatoid arthritis, age-related macular degeneration, and end-stage kidney disease over a total of 89,726 samples. Our method uses published prevalence data (as a function of clinical covariates) for each disease to estimate the LT parameters. The published prevalence data are an external source of information not utilized by the other statistical tests.

In these datasets, which include case-control and case-control-covariate designs, informed conditioning outperforms marginal logistic regression for 115 of the 157 known associated variants investigated (P-value = 1×10^−9^) with a 16% median increase in χ^2^ test statistic and a commensurate increase in power, attaining a substantial and highly statistically significant improvement in association statistics. We conclude that application of informed conditioning to future case-control-covariate ascertained and case-control ascertained association studies of these diseases, or other diseases with analogous effects of age, BMI, or other covariates on genetic risk, has the potential to substantially increase the power of disease gene discovery.

## Methods

### Liability threshold model

The model is defined by 
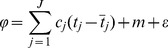
, where *ε* = *γg*+N(0,1), and an individual is a disease case (*z* = 1) if and only if *φ*≥0 and is a control otherwise (*z* = 0) [22]. Here *φ* is an unobserved underlying quantitative trait called the liability. The 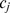 parameters quantify the effect of each covariate on the liability scale and *m* is an affine parameter that determines the disease prevalence at the covariate means 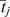 by 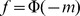, where 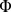 is the normal cumulative distribution function and 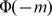 is P(x>−m). For diseases with prevalence less than 50% *m* will be negative. 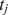 is the value of covariate *j*, 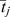 is the population mean of covariate *j*, *g* is the genotype of the candidate SNP (normalized to mean 0), *γ* is the effect size (equal to 0 under the null model) and N(0,1) is the standard normal distribution. The proportion of variance explained by covariate *j* on the liability scale is 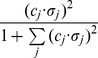 where 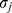 is the standard deviation of covariate *j*.

### Overview of method

Our method employs a three-step procedure. First, we fit the parameters 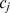 and *m* via a method (LTPub) that uses published prevalence information. Second, we compute the posterior mean residual liability 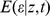 for each individual given the case-control status *z* and the values of the clinical covariates *t*. Missing covariates in cases are assigned the mean value of the covariate in cases and similarly for controls. Third, we perform linear regression of the posterior mean residual liability against the genotypes of the SNPs we wish to test while optionally incorporating additional covariates such as principal components (PCs), generalizing the EIGENSTRAT method [27]. Each of these steps is described in detail below. All methods described here are implemented in the LTSOFT software (see Web Resources). We note that there are important differences between our statistic and existing statistics such as those currently implemented in R (see Text S1 in File S1).

The approach is best illustrated by an example. We consider a simulated BMI-matched case-control-covariate type 2 diabetes (T2D) dataset. In T2D, prevalence is greater in the population of individuals with high BMI. Our toy example contains 3,000 cases and 3,000 controls, half with BMI = 24 and half with BMI = 35. (This gives a mean BMI of 29.5 and standard deviation of 5.5, similar to the real T2D studies analyzed below.) We first fit the parameters of the liability threshold model using published information on prevalence as a function of BMI. This procedure is described in detail below and gives a liability model *φ* = *c*(*t*−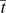)+*m*+*ε* where *c* = 0.08, *m* = −1.44, 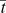 = 26.5, *ε* = *γg*+N(0,1). We choose *γ* = 0.1 and give *g* a minor allele frequency of 0.5. In this case *t* is BMI and 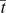 is the mean BMI. The parameter *c* is the coefficient of BMI in liability model. An individual is disease case if *φ*≥0 and a control if *φ*<0.

**Figure 1:**
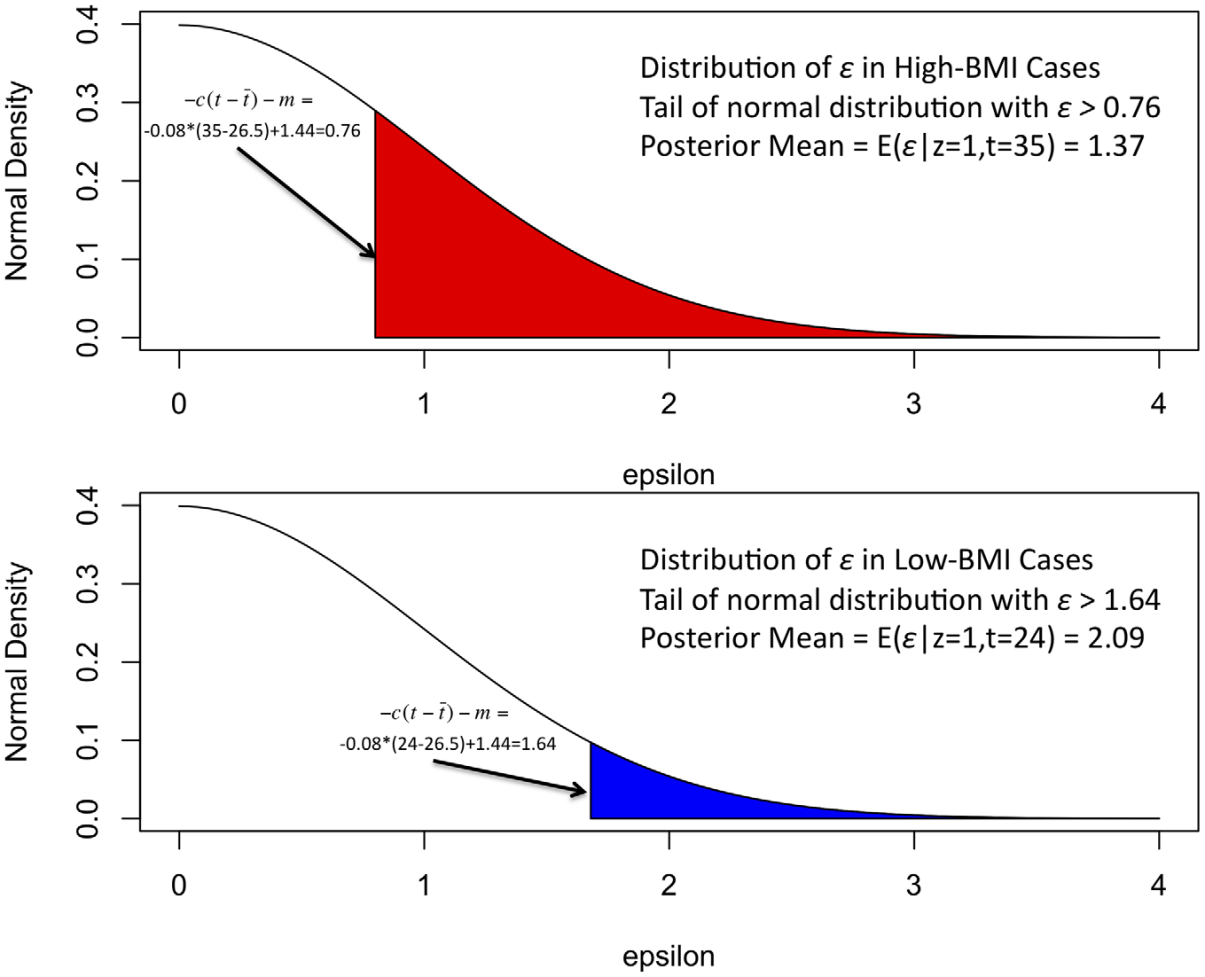
Illustration of liability threshold model: simulated T2D example. The posterior mean of *ε* for low-BMI and high-BMI cases is the expected value of *ε* given that it exceeds c(t−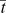)+m. High-BMI cases have a lower posterior mean relative to low-BMI cases since they require a smaller contribution from genetics to exceed the threshold in the liability threshold model.

We next compute the posterior mean value of the residual quantitative trait adjusted for BMI according to equations (1) and (2) below (Figure 1 and Table 1). Since the liability φ and *ε* are normally distributed, the posterior distribution of *ε* is the tail of normal. In Figure 1 this distribution is shown for the low-BMI and high-BMI cases. A BMI = 24 T2D case has a more extreme posterior mean value of *ε*, (2.09) than a BMI = 35 T2D case (1.37), because for BMI = 24 the lower contribution from BMI implies that a larger contribution from other factors (e.g. genetic factors) is needed to exceed the liability threshold. Similarly, a BMI = 35 T2D control has a slightly more extreme value (−0.36) than a BMI = 24 T2D control (−0.10), in order to stay below the liability threshold despite the higher contribution from BMI. In contrast, in standard linear regression all cases have the same value (e.g. 1) and all controls have the same value (e.g. 0).

**Table 1:** Illustration of liability threshold model: simulated T2D example.

	Posterior mean E(*ε|z,t*)	Allele frequency
Cases, BMI = 24	2.09	0.55
Cases, BMI = 35	1.37	0.53
Controls, BMI = 24	−0.10	0.50
Controls, BMI = 35	−0.36	0.49

Posterior mean value of residual quantitative trait *ε* (adjusted for BMI) as a function of BMI and case-control status. We also list allele frequencies specified in simulated genotype data.

We test a causal variant with minor allele frequency (maf) 0.5 in the population and an effect size on the liability scale of *γ* = 0.1 corresponding to an estimated odds ratio of 1.25 in the BMI = 24 cases and 1.16 in the BMI = 35 individuals (see Simulations). We compute association statistics for the liability threshold (LT) model using these posterior mean values (Table 1). Our LT statistic is a score test equivalent to a linear regression likelihood ratio test where the alternate likelihood is the likelihood of the posterior mean of the residual of the liability (E(*ε*|*z,t*)) under a linear regression model with an unconstrained genotype effect size. Under the null the genotype effect size is equal to 0.

In these simulations, the likelihood ratio test has an expected χ^2^(1 dof) = 30.3 (P = 3.7×10−8), which is genome-wide significant. It is notable that applying logistic regression (LogR) directly to case-control phenotypes produces a less significant statistic—either with or without conditioning on BMI, which has virtually no effect since cases and controls are BMI-matched. Logistic Regression of case-control status against genotype has an expected χ^2^(1 dof) = 27.9 (P = 1.3×10−7), and an expected χ^2^(1 dof) = 27.9 (P = 1.3×10−7) when using BMI as a covariate. Neither of these statistics is genome-wide significant. Studies with case-control-covariate ascertainment often attempt to match on a covariate, such as BMI in this example in order to prevent a loss of power that can come from stratified testing [13]. While it is true that the conditioned logistic regression test did not lose power relative to logistic regression, neither test obtained the power available to the LT statistic. This is because when there is no difference in the distribution of BMI between cases and controls logistic regression and other previous approaches [12], [13], [17], [26] will set the effect size of BMI to 0, while the LT statistic uses external epidemiological information to estimate the effect size of BMI.

### Estimating LT parameters from published data (LTPub)

We begin with published prevalence information over a range of values of clinical covariates. One means of finding the liability threshold parameters to minimize the normalized least-squares error
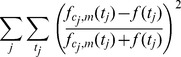
where 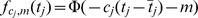 is prevalence at covariate value 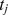 under the liability threshold model with parameters 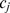 and *m*, and 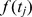 is the published prevalence at value 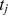. For example, prostate cancer is known to have prevalence 2%, 8%, 14% for individuals of age 60, 70, 80, respectively (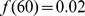, 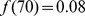, 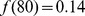) (see Text S1 in File S1). In this case, the parameters 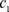 = 0.05 and *m* = −2.5 imply prevalence values of 2%, 7%, 16% for individuals of age 60, 70, 80 (based on standard normal probabilities for *ε*≥2.0, *ε*≥1.5, *ε*≥1.0 under the null model *γ* = 0, and a mean age of 50). In order to avoid the binary search procedure we transform the search from the disease scale to the liability scale minimizing
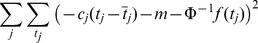
which can be solved analytically. We note that when *t* refers to age, the fact that some individuals will die before age *t_i_* is irrelevant to our computations, since the liability threshold model is defined for individuals who are alive at a given age *t*. The mean 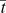 was chosen as the mean from the available prevalence data, and mis-specifying the mean has little effect (see Text S1 and Table S1 in File S1). For each disease studied, the source of prevalence data for each covariate is given in Text S1 in File S1.

When there are multiple covariates we treat them as independent but infer the parameters jointly. For example, in T2D we fit the parameters 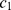 for age, 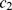 for BMI, and m (the affine term) simultaneously. We believe that this is a reasonable approximation so long as the covariates are only weakly correlated, as association statistics are robust to small deviations in model parameters (see below). When clinical covariates are highly correlated, treating them as independent will reduce power. It is possible to avoid this power loss by fitting the LT model with prevalence data for both covariates simultaneously (e.g. specifying the prevalence of T2D at all age/BMI pairs). For the datasets in this study, this was not necessary, as the squared correlation was less than 0.026 for all pairs of covariates.

### Association test using posterior mean value of underlying quantitative trait

The main idea is that instead of conducting an association test using case-control phenotype *z*, we use the posterior mean 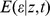 of the (unobserved) residual liability *ε*. Thus, 
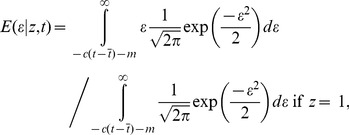
(1)

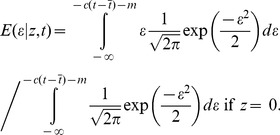
(2)When a study measures age at onset, or age and other covariates at onset, then the precise point at which the threshold is crossed is known, and 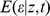 = 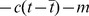 can be used. Our association statistic is a measure of association between genotype *g* and posterior mean residual liability 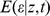 across samples. We treat 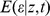 as a continuous variable and perform linear regression, computing the number of samples times the squared correlation between g and 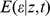, employing a generalized Armitage trend test [17], and generalizing EIGENSTRAT if PC covariates are also used [27], [28]. Although 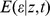 is not normally distributed, the use of linear regression as opposed to logistic regression is accepted practice in association studies [17], [27], [28]. Effect sizes are returned on the liability scale and these can easily be converted to odds ratios if desired (e.g. for meta-analysis) (see Text S1 in File S1).

We show below that this is equivalent to the Score test, which is also commonly used in genetic association studies [1], [29], [30]. We write the prospective likelihood as a function of effect size *γ* is
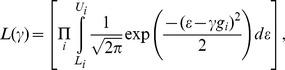
where 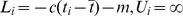 for cases and 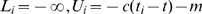 for controls. Thus, 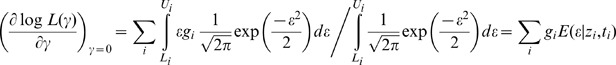
It follows that the Score statistic is equal to the square of 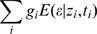 divided by its empirical variance, which is equivalent to the liability threshold statistic and has the correct null distribution. The retrospective likelihood is equal to this prospective likelihood (see Text S1 in File S1). We show below that this statistic is robust to parameter mis-estimation and maintains the correct null distribution (see Results).

## Results

### Simulations

We generalized the simulations from the toy case-control-covariate example for T2D above. These simulations used a BMI-matched design, which is a special case of case-control-covariate ascertainment. For each effect size *γ* between 0.00 and 0.15, we simulated independent datasets using the liability threshold model with a single clinical covariate with parameters *c* = 0.08 and *m* = −1.44. We refer to the clinical covariate as BMI, but the simulations apply equally to other clinical covariates. We assumed 3,000 cases and 3,000 BMI-matched controls, half with BMI = 24 and half with BMI = 35. We considered a SNP with allele frequency *p* = 0.50 in the general population. The estimated odds ratio of the SNP increases with the effect size, and the estimated odds ratio of individuals with BMI = 24 is larger than the estimated odds ratio of individuals with BMI = 35 for every non-zero effect size, consistent with Table 1. This is expected under the LT model since cases with BMI = 24 will generally need more risk alleles to reach *φ*≥0. For each value of *γ*, we simulated 1,000,000 independent datasets using *p*_case,24_, *p*_control,24_, *p*_case,35_, *p*_control,35_ based on the liability threshold model. Using these simulations, we evaluated power and false-positive rate. We also considered non-additive models, as well as the effect of mis-specifying the parameters of the LT model.

### Evaluation of power

We considered five different statistical tests: logistic regression (LogR) using case-control phenotype, LogR using case-control phenotype with BMI as covariate (LogR+Cov), a χ^2^(2 dof) test for main genetic effect and gene x BMI interaction (G+GxE) [18], [19], LogR comparing low-BMI cases to controls (LogRSub) [5], [20], and our association statistic (LT) using posterior mean residual liability from the LT model (see Methods). We note that the χ^2^(2 dof) statistic (G+GxE) is a likelihood ratio test comparing the null model of no main genetic effect and no gene x BMI interaction to the causal model with main genetic effect and gene x BMI interaction.

For each test, the average χ^2^ statistic is displayed in Table 2. We see that the LT statistic produces an average improvement of 8.8% in χ^2^ statistics compared to LogR. The improvement is a function of BMI distribution, effect size, disease prevalence, minor allele frequency, and study design. The G+GxE test loses power due to the extra degree of freedom. The LogRSub test performs nearly as well as the LogR test, showing that low-BMI cases contribute more power than high-BMI cases.

**Table 2:** Average χ^2^ statistics for LT versus other approaches in simulated data.

γ	LogR	LogR+Cov	G+GxE	LogRSub	LT	ORLBMI	ORHBMI
0.00	1.00	1.00	1.00	1.00	1.00	1.00	1.00
0.06	11.27	11.27	9.69	10.60	12.11	1.15	1.10
0.07	14.61	14.61	12.86	13.72	15.77	1.17	1.12
0.08	18.43	18.43	16.52	17.32	19.97	1.20	1.13
0.09	23.11	23.12	21.04	21.66	25.03	1.23	1.15
0.10	27.88	27.89	25.73	26.21	30.34	1.25	1.17
0.11	33.45	33.47	31.15	31.38	36.48	1.28	1.19
0.12	39.77	39.80	37.51	37.24	43.46	1.31	1.20
0.13	45.92	45.95	43.64	42.89	50.29	1.34	1.22
0.14	52.74	52.78	50.55	49.11	57.79	1.37	1.24
0.15	59.63	59.68	57.89	55.60	65.55	1.39	1.26

For each statistic we display average results across 1,000,000 simulations, for various effect sizes *γ*. All statistics are χ^2^(1 dof). Logistic regression with an interaction term (G+GxE) values been converted from χ^2^(2 dof) to the equivalent χ^2^(1 dof) value. At an effect size of 0 all statistics give the expected value under the null. OR LBMI is the odds ratio computed from cases with BMI = 24. OR HBMI is the odds ratio for cases with BMI = 35.

In addition to these five main tests we considered two additional tests: A χ^2^(1 dof) statistic, which compares the null model of main genetic effect only to the causal model with main genetic effect and gene x BMI interaction, and is equal to the difference between G+GxE and LogR statistics; a case-only logistic regression comparing BMI = 24 to BMI = 35 [21]. These gene-environment interaction tests had χ^2^(1 dof) statistics less than 5.0 for all effect sizes and are not considered further. Another approach, probit regression [31], uses an underlying model which is equivalent to the liability threshold model. However, probit regression does not account for disease prevalence, the effect sizes of covariates estimated from the epidemiological literature, or the ascertainment scheme used by the study and therefore produces very different statistics from the LT model (see Text S1 in File S1). Probit and linear regression gave similar results to logistic regression over all simulations and real datasets. This result was obtained both with and without covariates.

**Figure 2:**
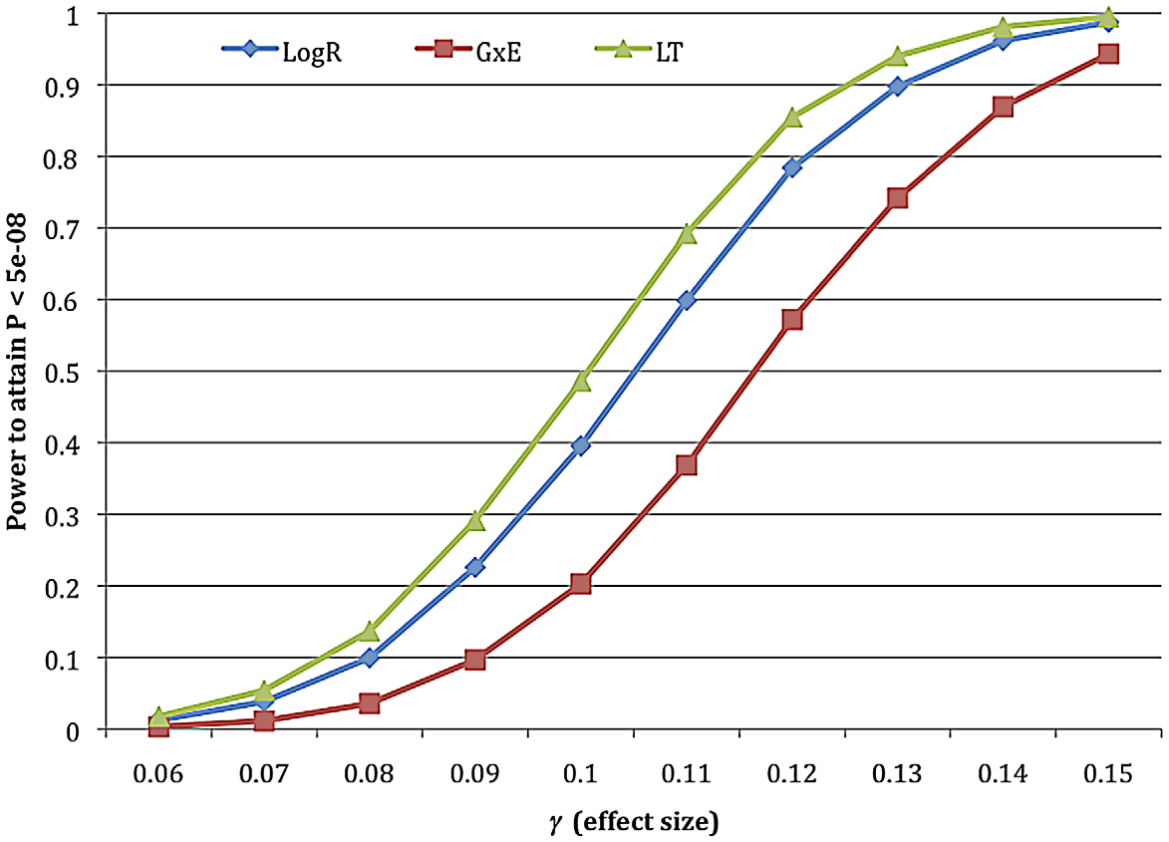
Power calculations for LogR, G+GxE, and LT approaches in simulated data. For each statistic we display power to attain P<5**×**10^−8^ based on 1,000,000 simulations of 3000 cases and 3000 controls, for various effect sizes *γ*. The increase in power (ratio of y-axis values) for LT versus LogR is 22.8% for *γ* = 0.1, and 23.0% when computing average power across all values of *γ*. For γ = 0 the power was 5.0% for all statistics when the P-value threshold is 0.05. G+GxE performs worse due to an extra degree of freedom.

Average χ^2^ statistics are useful for comparison purposes, but do not provide a formal assessment of power. We also performed power calculations, computing the proportion of 1,000,000 simulations achieving the conventional GWAS cutoff for significance at 5% level following correction for multiple testing of P<5×10^−8^. Results for a subset of methods are displayed in Figure 2, indicating a 23% improvement in power for the LT statistic. In all simulations the percent improvement in power is substantially larger than the percent improvement in average χ^2^ statistic. We caution that these results will vary as a function of the ascertainment of BMI in the study. Furthermore, for any choice of ascertainment strategy, these results may overstate the prospects for improvement in real data, since simulated data and association statistics were based on the same model and model parameters.

We repeated the above experiments under a range of ascertainment schemes (random, case-control, case-control-covariate) and effect sizes (see Text S1 and Table S2 in File S1). In all experiments the LT statistic matched or outperformed all of the other statistical tests while maintaining the correct null distribution. For randomly ascertained studies, there is no induced correlation between genotype and clinical covariate and we do not expect or observe an improvement in our method over the others [7]. In many cases conditioning on BMI significantly decreased power. Under case-control ascertainment strategies, covariates correlated with case-control status will also be correlated with associated genotypes [10]. Conditioning on these covariates can therefore introduce biases and reduce power [9], [10], [11] as a function of covariate effect size and disease prevalence (see Text S1 and Table S3 in File S1). Our method performs better than previous approaches including [12] (see below), because covariate effect sizes can be estimated more accurately using external epidemiological information. Matched case-control-covariate designs, in which covariates are matched in some proportions between cases and controls, may prevent conditioning from having any effect in existing methods. Since the LT statistic uses information from external epidemiological literature it can still produce an improvement.

### False-positive rate and correct null distribution

To investigate the properties of the LT statistic under the null we computed the mean value in the simulations above when *γ* = 0.0. As seen in Table 2 this has the correct value of 1.00. In addition it has the correct median, with 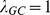.00, 5.00% of tests with P-value<0.05 and 1.00% of tests with P-value<0.01. We applied Kolmogorov-Smirnov test [31] to determine if the LT statistic differed significantly from a χ^2^ (1 dof) distribution. The two-tailed K-S test of the full distribution was not significant (P-value = 0.34), nor was the K-S test restricted to the tail where the LT statistic had χ^2^>3.84 (P-value = 0.21). In order to further investigate the extreme tail of the distribution we ran 10^8^ tests under the null and verified that 98 of the 10^8^ tests (10^−6^) had a P-value<10^−6^. The LT statistic is a score test when the parameters are estimated correctly and will therefore have the correct null distribution. We investigated the properties of the LT statistic when the parameters were severely mis-estimated and found no inflation (see Text S1 in File S1). Furthermore, since the LT statistic is an ATT test between *g* and the posterior mean of the residual of the liability 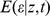, it will not have an inflated false-positive rate provided that 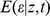 does not have heavy tails or extreme heteroscedasticity [32]. 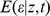 is the area under the tail of a normal distribution and will therefore not have these properties provided that the clinical covariate does not.

### Logit disease model

The LT statistic assumes the same model used to generate the data in the above experiments, and its increase in performance over other methods may be driven by this fact. To examine this possibility we conducted case-control study simulations under a logit model as opposed to liability threshold model of disease used above. We also performed simulations in which the LT parameters were estimated from simulated epidemiological summary statistics. In all cases, the LT statistic continued to outperform the other methods by a similar margin (see Text S1 and Table S6 in File S1). We conclude that leveraging external epidemiological data and not the similarity of the generative model to the tested model drives the increase in power.

### Non-additive models

Our above simulations examine a range of alternatives consistent with additivity on the liability scale. While data and theory suggest that additivity explains most of the genetic variance for a range of phenotypes [33], many researchers are interested in a wider range of models involving gene x covariate interaction on the liability scale. We simulated additional datasets in which we added a positive or negative interaction term (see Text S1 and Table S4 in File S1) and found that the relative performance of the LT statistic depends on the direction of the interaction. Negative interaction, such as the recently discovered coffee-GRIN2A interaction in Parkinson's disease [34], increases the power of LT. Positive interaction, such those recently found for smoking and lung cancer [35] decreases the power of LT (see Table S4 in File S1). The G+GxE test outperformed the other statistical tests in most of these simulations, although the LT statistic performed better than G+GxE when the interaction term was negative. Averaging across gene x covariate interactions in either direction, LT outperformed LogR. This supports the use of LT instead of LogR, even accounting for the possibility of gene x covariate interaction on the liability scale.

### Other statistical tests

Adjustment for informative covariates is not unique to genetics and the problem of estimation from case-control data has received considerable attention [10]. propose a weighted logistic regression method (inverse-probability weighting) in the case of conditioning on clinical variables in case-control ascertainment studies [9]. also offer an efficient estimator for case-control ascertainment studies in order to account for ascertainment-induced biases. In the case of inverse-probability weighting, unbiased effect sizes are indeed obtained, but it under-performed relative to the LT statistic in simulations, with a 7% lower χ^2^ than the LT statistic in the simulations from Table 2 when *γ* = 0.1 [12]. propose using a retrospective likelihood to address case-control ascertainment issues when conditioning on a covariates and implement a semi-parametric test to incorporate the clinical covariates. In our case-control simulations, the LT statistic outperformed this method (see Text S1 in File S1). In the case of case-control-covariate designs this semi-parametric test, as well as other previous approaches [13], [26], are not expected to improve power because they can not leverage external epidemiological literature describing the clinical covariates.

### Mis-specification of model parameters

To investigate the sensitivity of the LT statistic to mis-specification of model parameters, we performed additional simulations in which we assumed model parameters that were different from those used to simulate the data. We concluded that the LT statistic is robust to deviations in model parameters (see Table S1 in File S1). However, only analyses of empirical data can determine whether the liability threshold model provides a good fit to real diseases.

### Real datasets

#### Estimation of model parameters for real diseases

We estimated parameters for each of the diseases using published prevalence data as a function of the relevant covariates. For example, for T2D we used prevalences 2%, 3%, 5%, 8%, 13%, and 24% for BMIs 18, 21.5, 24.5, 27.5, 30.5, 35 respectively. Using these data we fit the liability threshold model parameters so as to minimize the squared error between the expected thresholds and those specified by the model(see Methods). The values used to fit the parameters and the sources of this information are given in Text S1 in File S1. The inferred parameter values for each disease studied are displayed in Table 3. These studies include both case-control-covariate ascertainment as well as case-control ascertainment strategies (see Table 4).

**Table 3:** Inferred covariates and effect sizes on the liability scale.

Disease	%VarianceExplained	LT Model for φ
T2D (Metabo)	BMI = 14%, age = 6%	0.08*(BMI-26.5)+0.029*(age-50)-1.38
	BMI = 15%	0.08*(BMI-26.5)-1.44
	age = 9%	0.029*(age-50)-1.28
T2D (MEC)	BMI = 14%, age = 4%	0.08*(BMI-26.5)+0.029*(age-50)-1.38
	BMI = 15%	0.08*(BMI-26.5)-1.44
	age = 5%	0.029*(age-50)-1.28
PC	age = 14%	0.049*(age-50)-2.49
LC	age = 2%,smoking = 76%	0.03*(age-50)+2.6*(smoking-0.25)-3.06
	age = 17%	0.04*(age-50)-3.30
	smoking = 51%	2.04*(smoking-0.25)-2.37
BC	age = 8%	0.032*(age-50)-2.26
RA	age = 6%, sex = 2%	0.022*(age-50)+0.32*(sex-0.5)-2.46
	age = 6%	0.022*(age-50)-2.46
	sex = 2%	0.32*(sex-0.5)-2.34
ESKD	age = 15%	0.02*(age-50)-2.08
AMD	age = 17%, BMI30 = 5%	0.03*(age-50)+0.61*(BMI30-0.30)-2.00
	age = 11%	0.04*(age-50)-2.10
	BMI30 = 6%	0.35*(BMI30-0.30)-1.72

LT model is the liability threshold model for each disease with parameters estimated using the LTPub method. For diseases with multiple covariates, models with all covariates and each covariate separately are given. %Variance Explained is the fraction of variance explained on the liability scale in the study data for each of the covariates in each of the diseases when all covariates are used in the model, and is specific to the distribution of covariates in each particular study. BMI30 is a binary variable, which is 1 if an individual's BMI is greater than 30 and 0 otherwise. Type 2 diabetes (T2D), prostate cancer (PC), lung cancer (LC), breast cancer (BC), rheumatoid arthritis (RA), end-stage kidney disease (ESKD), and age-related macular degeneration (AMD).

**Table 4:** Summary information for all datasets.

Disease	Ascertainment	Cases	Controls	SNPs	ORL>ORH	LTPub>LogR
T2D (Metabo)	Case-Control-Covariate	5051	3529	47	37	37
T2D (MEC)	Case-Control-Covariate	6142	7403	19	15	16
PC	Case-Control-Covariate	10501	10831	39	32	30
LC	Case-Control-Covariate	6952	6661	16	13	12
BC	Case-Control-Covariate	9619	12244	20	12	11
RA	Case-Control	5024	4281	21	16	15
ESKD	Case-Control	1030	1025	1	1	1
AMD	Case-Control-Covariate	473	1103	2	2	2
SUM	n/a	37840	40416	165	128	128

ORL>ORH is the number of SNPs in which the odds ratio of low risk cases (e.g. low-BMI) is greater than then odds ratio computed from the high risk group (e.g. high-BMI). LTPub>LogR is the number of SNPs in the dataset for which LTPub exceeded the LogR statistic. There are 9 SNPs shared between the two T2D sets. In total there are 157 unique SNPs and 115 unique SNPs with LTPub>LogR. Type 2 diabetes (T2D), prostate cancer (PC), lung cancer (LC), breast cancer (BC), rheumatoid arthritis (RA), end-stage kidney disease (ESKD), and age-related macular degeneration (AMD).

#### Type 2 diabetes datasets

We applied informed conditioning to a case-control-covariate ascertained dataset of 5,051 T2D cases and 3,529 controls from three Swedish cohorts (the Malmo Preventive Project, Scania Diabetes Registry, and Botnia Study) [16] genotyped on the Metabochip [36]. This study oversampled low-BMI cases and younger cases, but did not explicitly match cases and controls for BMI or age. The genotyped SNPs include 47 SNPs identified by previous type 2 diabetes genome-wide association studies (GWAS) [1]. T2D and BMI is a particularly compelling example for analysis with the LT statistic, as we report in Table S9 of ref. [1] that 23 of 29 T2D SNPs have higher effect size for low-BMI versus high-BMI cases (P-value = 0.0003; average odds ratios 1.182 versus 1.128, P-value for heterogeneity not significant for most individual SNPs). (Also see [37], 29 of 36 T2D SNPs have higher effect size with average odds ratios 1.13 versus 1.06 for low-BMI versus high-BMI cases). Individuals are clinically diagnosed with T2D if their fasting glucose exceeds a specific level. The similarity between an underlying liability and fasting glucose exceeding a threshold further motivates the use of an LT model to analyze T2D.

We compared association statistics over these T2D data using four approaches: LogR, LogR+Cov, logistic regression with an interaction term (G+GxE), and LT. Logistic regression without high-BMI cases (LogRSub) was not included since it contains strictly fewer individuals and its performance is not expected to exceed LogR. The G+GxE test underperformed relative to other methods in all datasets due to its extra degree of freedom. This is expected since the SNPs were discovered with a marginal test, and are therefore less likely to have gene x covariate interactions on the liability scale. Results are displayed in Table 4, Table 5, and Table S8 in File S1 and we see that the sum of χ^2^ statistics across all loci is 51% higher for LT than LogR. As expected under an LT model, the odds ratios computed from individuals with low BMI are greater than those computed from individuals with high BMI. The T2D LT models also use age as a covariate and in the LTPub estimation method age and BMI were fit jointly (see Methods). We reran the LTPub estimation fitting BMI and age separately and found the improvements over LogR to be 32% and 18% respectively.

**Table 5:** Summary statistics across all datasets.

Disease	LTPub	LogR	LogR+Cov	LTPub vs LogR
T2D (Metabo)	369.7	244.05	252.23	+51%
T2D (MEC)	402.86	320.08	400.89	+26%
PC	1912.88	1787.61	1844.40	+7%
LC	416.95	359.64	331.28	+16%
BC	395.16	390.86	386.83	+1%
RA	511.31	470.91	466.11	+9%
ESKD	188.38	137.80	134.70	+37%
AMD	185.6	159.38	110.33	+16%

The sum of each of the test statistics across all of the SNPs in each of the diseases. LTPub vs LogR is the % increase of LTPub compared to LogR. It has a median value of 16%. Type 2 diabetes (T2D), prostate cancer (PC), lung cancer (LC), breast cancer (BC), rheumatoid arthritis (RA), end-stage kidney disease (ESKD), and age-related macular degeneration (AMD).

It is of interest to include non-European ancestries in studies of T2D, because non-Europeans have higher T2D risk [38], [39]. We examined the performance of the same six statistics over of 6,142 cases and 7,403 controls genotyped at 19 known associated SNPs from the Multiethnic Cohort (MEC) (African Americans, Latinos, Japanese Americans, Native Hawaiians, and European Americans) [38]. A potential concern is that risk SNPs identified in Europeans may not be associated in other populations due to different LD patterns, however, previous analyses have demonstrated that these 19 SNPs are consistently associated to T2D in all MEC ancestries [38]. Results are displayed in Tables 4–5 and Table S9 in File S1. We see that application of LT attains 26% higher χ^2^ statistics than LogR. We reran the LTPub estimation fitting BMI and age separately and found the improvements over LogR to be 20% and 3% respectively.

The Metabochip study included a large number of low-BMI cases as part of their ascertainment strategy whereas the MEC study ascertained randomly with respect to BMI. Including low-BMI cases increases the power of the Metabochip study since odds ratios estimated from the population of low-BMI individuals will be larger [16] (Table S9 of ref. [1]). This is predicted by the liability threshold model since low-BMI cases require additional factors (i.e. genetic factors) to exceed the threshold. In our simulations (see Text S1 and Table S2 in File S1) the improvement of LT over LogR was even greater with this ascertainment strategy than it was in a standard case-control ascertainment strategy. Thus, this strategy gives even greater performance of the LT statistic relative to LogR because the low-BMI cases will be up-weighted relative to the high-BMI cases. This is likely the cause of the better performance of LT in Metabochip compared to the MEC dataset.

For each T2D dataset, we simulated 100,000 datasets with the same sample sizes, covariates, and case-control status as the real datasets. We simulated a causal variant with effect size 0.1 and minor allele frequency 0.1 under the LT model for T2D and computed statistics for LT and LogR. The percent improvements were 40%
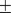
21% for Metabochip and 22%
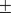
6% for MEC similar to those in the real datasets (see Table S5 in File S1).

#### Prostate cancer dataset

We applied informed conditioning to a case-control-covariate ascertained dataset of 10,501 prostate cancer cases and 10,831 controls (with 7 of 8 cohorts age-matched) from the NCI Breast and Prostate Cancer Cohort Consortium (BPC3) that were genotyped at 39 SNPs identified by previous prostate cancer GWAS [40]. We previously reported that 32 of 39 SNPs had a higher odds ratio for early-onset cases versus late-onset cases (Table S3 of ref. [40]), which is unlikely to be due to chance (P<0.0001) and motivates the question of whether informed conditioning of prostate cancer might increase power.

We compared association statistics using four approaches: LogR, LogR with age as covariate, logistic regression with an interaction term (G+GxE), and LT. As was the case for T2D, G+GxE underperformed relative to the other methods due to its extra degree for freedom. Results are displayed in Tables 4–5 and Tables S10–S11. We see that application of LT attains 7% higher sum of χ^2^ statistics than LogR and that the odds ratios computed from early-onset cases are greater than those computed from late-onset cases. Including study cohort as a covariate had no significant effect on these tests. The age information in this study is age at onset and we therefore repeated the analysis using 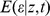 = 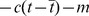 in cases (see Methods). This increased the sum of χ^2^ statistics from 1912.88 to 1925.65.

We repeated the analysis computing association statistics separately for each of the eight BPC3 cohorts and performing a meta-analysis across cohorts using inverse variance weighting to combine test statistics [41]. Results were broadly similar, with a 7% increase in the sum of χ^2^ statistics of LT compared to LogR. However, one difference is that LogR with age as covariate produced a 1.3% increase in χ^2^ statistics in the combined analysis (both with and without study as a covariate) but a 2.3% decrease in χ^2^ statistics in the meta-analysis. We sought to understand this difference by comparing performance separately for each cohort. We determined that LogR with age as covariate performs similarly to LogR if cases and controls are age-matched, performs worse than LogR if controls are much younger, slightly older or much older than cases, but performs better if controls are slightly younger than cases—as in the HPFS cohort and in the combined analysis. LogR with age as covariate performs better in the latter case because age-adjusted case-control phenotype has a more extreme value in younger cases than in older cases, mimicking the posterior mean quantitative trait phenotype used in the LT statistic. The effect of conditioning covariate in LogR is a complex function of ascertainment strategy, effect size, and the distribution in the cohort, and should not be viewed as a method that improves power in the general case [9], [10], [11].

For the prostate cancer dataset, we simulated 100,000 datasets with the same sample size, covariates, and case-control status as the real dataset. We simulated a causal variant with effect size 0.07 and minor allele frequency 0.05 under the LT model and computed statistics for LT and LogR. The percent improvement was 6%
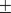
3%, similar to that in the real dataset (see Text S1 and Table S5 in File S1).

#### Other datasets

In addition to T2D and prostate cancer, we examined lung cancer [42], [43] with age as a covariate, breast cancer [44], [45] with age as a covariate, rheumatoid arthritis [46] with age and sex as covariates, age-related macular degeneration [47] with age as a covariate, and end-stage kidney disease [48] (ESKD) with age as a covariate (Tables 4–5 and Tables S5,S11–S15). The breast-cancer, lung-cancer, and age-related macular degeneration studies are matched case-control-covariate ascertained, and the rheumatoid arthritis and ESKD studies are case-control ascertained. The parameters for the LTPub model were set according to published prevalence studies for the appropriate covariates and diseases (see Text S1 in File S1). In each case we compared the relative performance of the LT statistic to the standard association test statistics over known associated SNPs with results presented in Tables 4–5 and Tables S11–S15. The LT statistic improved 16% for lung cancer, 1% for breast cancer, 9% for rheumatoid arthritis, 37% for end-stage kidney disease, and 16% for age-related macular degeneration (see Table S5 in File S1). Across all datasets 115 out of 157 SNPs had higher odds ratios in the low risk group as expected from the LT model. The age information in the breast cancer study is age at onset and we therefore repeated the analysis using 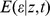 = 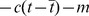 in the cases (see Methods). This decreased the sum of χ^2^ statistics from 395.16 to 393.39.

Averaging across the eight datasets analyzed, the LT approach we propose attained a median improvement of 16% and mean improvement of 20% as compared to the commonly used LogR method, with an improvement for 115 of 157 SNPs (P-value = 1×10^−9^). To show that relative improvement of LT is not solely due to SNPs with large values of LogR, we computed the sum of LT and LogR for the SNPs in the lower 50% of LogR for each disease excluding the single SNP of ESKD. The LT statistic had a 15% median improvement and an 18% mean improvement over LogR for these lower 50% SNPs. We also ran permutations to show that the gains of the LT relative to LogR require the correct covariate information and that genotype and covariate are correlated for known loci, as predicted by the liability threshold model (see Text S1 in File S1) and any penetrance model where genotype and clinical covariate affect outcome [10].

T2D and lung cancer are both affected by clinical covariates (BMI and smoking status) that are partly genetically driven. In such instances, LT modeling of the covariate will generally increase power to detect SNPs whose primary association is to the disease, and reduce power to detect SNPs whose primary association is to the covariate with secondary association to the disease. In light of this, LT modeling of the covariate is our recommended strategy, since SNPs whose primary association is to the covariate are best discovered via separate studies of association to the covariate trait. Following this strategy, we used both BMI and age as covariates for T2D. We note that the T2D SNPs tested include one locus (FTO) which has a primary association to BMI with induced secondary association to T2D [49]. As expected, LT performed poorly at FTO SNPs (Table S8, S9 in File S1). We elected to include FTO SNPs in our computation of % improvement in order to avoid overstating our results, but we believe it would be technically appropriate to exclude these SNPs from this computation, since they would be best discovered by a separate study of association to BMI.

In the case of lung cancer, if the goal is to identify lung cancer SNPs (rather than smoking SNPs) we recommend including both age and smoking as covariates. However, our task of evaluating the LT model for lung cancer was complicated by the fact that many known lung cancer loci have a primary association to smoking with a secondary (less statistically significant) association to lung cancer [50], [51]. Therefore, we conservatively report the improvement for using age as a covariate only. However, we believe it would be technically appropriate to exclude smoking SNPs from the computation and report the larger improvement for age and smoking as covariates for the remaining SNPs. Therefore, we reran the lung cancer data on the subset of five SNPs that do not have a primary association to smoking status, and fit both age and smoking status with LTPub to get φ = 0.030*(age-50)+2.59*(smoking-0.25)−3.06, where smoking is status as a smoker or non-smoker. Age described 2% of the variation on the liability scale and smoking status described 76%. The improvement of LT over LogR was 30% for age and smoking, 27% for age only, and 11% for smoking status only.

### False-positive rate and correct null distribution

For each disease we permuted the genotypes of the individuals, keeping the case-control and covariates fixed 100,000 times. We reran the LT statistic on each permutation using the same LTPub parameters for each disease as above, and verified that LT had the appropriate 5% type 1 error rate at each SNP and 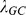 = 1.00. Additionally, we computed LT statistics on the complete Women's Genome Health Study (WGHS) age-related macular degeneration GWAS dataset of 339,596 SNPs [52]. There were 5.00% of tests with P-value<0.05 and 1.02% for P-value<0.01. Furthermore the Kolmogorov-Smirnov test [31] with a χ^2^(1 dof) distribution was not significant (P-value = 0.26), nor was the K-S test restricted to the tail with LT χ^2^>3.84 (P-value = 0.15).

## Discussion

We have shown that informed conditioning on clinical covariates in association studies with case-control-covariate or case-control ascertainment yields a substantial increase in power in the simulations and empirical datasets analyzed here. The gain in power varies across diseases and is a function of the proportion of variance on the liability scale explained by the covariate(s), the disease prevalence, and the ascertainment strategy. We note that the increase in power will often exceed the increase in χ^2^ statistics. For example, a GWAS with 5000 cases and 5000 controls has 43.7% power at P-value threshold 5×10^−8^ to detect a SNP with a minor allele frequency of 20% and an odds ratio of 1.2. The power increases to 59.8% (a>36% increase in power, in the sense that >36% more variants will be discovered) when increasing χ^2^ statistics by 16%, which is similar to the median increase in χ^2^ statistics that we observed in our empirical studies. Additional significant gains in power, particularly under the LT approach, are possible by collecting cases at phenotypic extremes [16], [53], [54], [55], [56], [57], taking care to check for SNPs associated with covariate as opposed to the disease [10]. The use of genetic covariates in the LT framework may also significantly increase the power of association studies. In that context we recommend a different method for estimating LT model parameters [26]. The LT approach is also applicable to data obtained from high throughput sequencing studies [16].

Thus, there is a very strong motivation for applying the approach we have described to type 2 diabetes, prostate cancer, lung cancer, age-related macular degeneration, and end-stage kidney disease (for which the LTPub parameters in Table 3 can be used), as well as for other diseases with analogous effects of clinical covariates on genetic risk (for which LTPub can be used to estimate parameters). For T2D and prostate cancer alone, we identified 29 recent studies published in *Nature Genetics* (see Text S1 in File S1) that would benefit from application of our method. Notably, our empirical improvements are in line with the improvements that would have been expected based on SNP and covariate effect sizes in these same datasets. In the case of diseases with genetically driven covariates (e.g. BMI in T2D) we recommend using all available covariates unless the goal is to identify SNPs whose primary association is to the covariate. There are many other diseases with important clinical covariates where informed conditioning may prove useful [58], [59], [60]. Recent studies of age-related macular degeneration [61] and gout [62] found increased odds ratios estimated from younger cases and genetic associations to age of onset, which is consistent with the LT model.

We caution against the use of standard conditioning approaches (LogR+Cov) in case-control ascertained studies, which can increase or decrease power as a function of covariate effect size and disease prevalence [8], [10], [26]. The relationship between modeling disease on the liability threshold and dichotomous scale has been examined by [23] as well as [24], [25] in the context of computing the area under the receiver operator curve (AUC), estimating risks, and the distribution of disease in a population. A recent study of Clayton has examined the use of covariates in case-control ascertained association studies and shown that a reweighting method (such as ours) can increase power [13]. This paper discusses the issue of power loss from conditioning [8] in logistic regression and states, “the loss of power resulting from the use of stratified tests can be avoided by matching in the design of case-control studies”. We have shown that by including information from external epidemiological information, it is possible to not only avoid a power loss, but to achieve substantial power gain in matched case-control-covariate studies. The paper also states, “the strategy of ignoring other known disease susceptibility loci and risk factors when testing for new associations with complex disease, for example in genome-wide association studies, is justifiable, but only when effects combine additively on the logistic scale.” While ignoring other risk factors is justifiable when testing under a retrospective logit model, we have demonstrate here, that for diseases with non-infinitesimal prevalence, and assuming gene environment independence, it is possible to achieve power gains even when the disease model is additive under a logit model. This was also shown in the work of [12] under a prospective logit model. We discuss additional approaches to analysis case-control ascertained data in Text S1 in File S1.

We designed the LT method for effects that are additive on the liability scale, which are hypothesized to account for the majority of genetic variation across a range of complex phenotypes [33]. We have shown empirically that it also behaves well under the standard additive logit model. In the presence of gene x covariate interaction it alternatively loses or gains power depending upon the direction of the interaction, but the method's increase in power does not rely on the presence of interaction. When interaction is present, other methods, such as logistic regression with an interaction term (G+GxE), may be more powerful. However, the LT statistic outperformed commonly used tests such as LogR on average in simulations of gene x covariate interaction (Table S4 in File S1), and remains our recommended approach after accounting for the possibility of such interactions. We note that when there is *no* true gene x covariate interaction on the liability scale, but individuals are ascertained based on phenotype, there will be an induced correlation between clinical covariate and genotypes associated with phenotype. Furthermore, there may be evidence of GxE interaction on the odds ratio scale and we therefore caution against inferring a biological mechanism of interaction when the data are consistent with additivity on the liability scale.

Meta-analysis is easily handled in the context of the liability threshold framework. Summary statistics are typically combined using odds ratios and standard errors. The LT statistics returns effect sizes on the liability scale and standard errors. Since these are easily converted to odds ratios (see Text S1 in File S1), and a standard inverse variance weighting can be used to combine results on either scale to generate a meta-analysis statistic. Furthermore, since odds ratios are a function of covariate ascertainment (e.g. if young cases are oversampled), meta-analysis on the liability scale maybe able to provide more robust estimates of effect size. Replication of results works as normal, additional cases and controls are collected, genotyped, and tested for association. If covariate information is not available in the replication set a standard LogR test is used.

The LT statistic uses covariates to increase power. We assume that the LT model parameters estimated from epidemiological data, as well as the values of the covariates measured in the study, are reasonably accurate. Under inaccurate estimation of model parameters our method will have reduced power relative to its power with accurate model parameters, but it will still have the correct null distribution. In simulations, mis-specifying the parameters by a moderate amount produced almost no change in power and mis-specifying the parameters by a large amount (up to 100%) still performed at least as well as logistic regression with no conditioning in all cases examined (see Text S1 in File S1). Accounting for uncertainty in the data from the epidemiological literature may further improve the increase in test statistic beyond the 16% observed in this analysis. Additional covariates (e.g. principal component covariates) may be needed to prevent false positives [27], [63]. These are easily handled by the LT statistic and included in the linear regression after the posterior means are computed (see Text S1 in File S1). When a genetically driven covariate is correlated to the phenotype (e.g. BMI in T2D), including that covariate in the LT model will alter the power to find SNPs related to phenotype through the covariate (see Text S1 in File S1). When using extreme sampling of a covariate (e.g. BMI in the T2D Metabochip study), there exists the theoretical possibility of misclassifying a covariate (BMI) association as a phenotype (T2D) association [49], because the posterior means may be correlated with BMI. Our recommendation is to check this by testing for association to the covariate (BMI) explicitly. A more conservative approach is to use BMI as a covariate after posterior means are computed, but some of the increase in power may be lost.

When conducting an association study where known clinical factors alter disease risk, the gain in power of the LT statistic is function of the number of individuals with available covariate information. For example, in the DIAGRAM dataset all 31 cohorts had BMI information and 20 had age at diagnosis information, thus the gain in power possible from the LT method will be nearly maximal [1]. If the increase in χ^2^ is 16%, then an individual with a covariate provides the same power as 1.16 individuals with no covariate. Researchers should therefore carefully weigh the cost of collecting covariates when designing studies since it may provide a more cost effective way to substantially increase power than genotyping more individuals.

In cross-sectional studies when data are randomly ascertained with respect to both case-control status and clinical covariate, the LT statistic and LogR+Cov are expected to perform similarly and our recommendation is to use LogR+Cov. In case-control studies of high prevalence diseases when clinical covariates are randomly ascertained, but cases are oversampled relative to their prevalence in the population, the LT statistic will slightly outperform LogR+Cov and our recommendation is to use the LT statistic. In case-control diseases of low prevalence, or in case-control-covariate studies when clinical covariates are non-randomly ascertained the LT statistic will substantially outperform LogR+Cov (which may often lose power relative to LogR) and our recommendation is to use the LT statistic. As described above, the LT statistic also outperforms other methods. In summary, informed conditioning on clinical covariates has a large potential to increase the power of case-control association studies and identify new risk variants.

### Web resources

LTSOFT software: http://www.hsph.harvard.edu/faculty/alkes-price/software/

## Supporting Information

File S1 Supporting information.
(DOC)
