# Supplementary material for: Informed Conditioning on Clinical Covariates Increases Power in Case-Control Association Studies
Source: PLoS Genet. 2012 Nov 8;8(11):e1003032. doi: 10.1371/journal.pgen.1003032 (PMC3493452; doi:10.1371/journal.pgen.1003032)
Supplement: File S1 — Supporting information. (DOC) [file pgen.1003032.s001.doc]

Supplementary Text S1

Prevalence and covariate data. Age and prevalence for prostate cancer was obtained at <http://seer.cancer.gov/csr/1975_2007/index.html>. Age and BMI prevalence for T2D data comes from [http://diabetes.niddk.nih.gov](http://diabetes.niddk.nih.gov/), and Lipton et al.[1], and Narayan et al[2]. Age and smoking information for lung cancer comes from http://seer.cancer.gov/csr/1975_2007/results_merged/sect_15_lung_bronchus.pdf and Godtfredsen el al.[3]. We used smoker versus non-smoker including former-smoker with non-smokers. Cigarettes per day and separating former-smokers from non-smokers produced similar results. Age information for breast cancer, was obtained at http://seer.cancer.gov/csr/1975_2007/results_merged/sect_04_breast.pdf.

Age information for rheumatoid arthritis, is from Stolt et al[4]. Age and BMI information for age-related macular degeneration is from Friedman et al[5]. Age information for end-stage kidney disease is from Kiberd et al[6]. The specific values used by LTPub to fit the LT model parameters are included in the LTSOFT software package.

LT and Probit Regression

A similarity between LT and probit regression is that they both assume an underlying normally distributed phenotype with cases being individuals exceeding 0. However, in the case of ascertained data, like the case-control studies above, probit regression will estimate different a different affine term and covariate effect sizes from LT resulting in a loss in power, with similar performance to LogR+Cov. For cross-sectional studies, in which cases and controls are randomly ascertained, probit regression with covariates and LT are equivalent in the sense that the expected covariate effect sizes and the expected thresholds are the same. Note that LT is not equivalent to probit regression with an offset parameter and the R code r=glm(trait~1, offset=c*t, family=binomial(link=probit))$residuals;fit=lm(r~genotypes) will often perform much worse than our statistic as it does not account for ascertainment via the prevalence term *m*.

Prospective Likelihood of the LT model

The LT statistic uses clinical covariate effect sizes and an affine term estimated from the epidemiological literature. Given these terms, the retrospective likelihood is equal to the prospective likelihood. We showed that the prospective likelihood is $gE(\varepsilon|z,t)$ (see Methods). The retrospective likelihood is:

$$\begin{matrix} \Pr(g|z,t)=\frac{\Pr(z|g,t)\Pr(g|t)}{\sum_{g} \Pr(z|g,t)\Pr(g|t)} \\ \frac{\partial}{\partial\gamma}\log(\Pr(g|z,t))= \\ \frac{\partial}{\partial\gamma}\log(\Pr(z|g,t))+\frac{\partial}{\partial\gamma}\log(\Pr(g|t))-\frac{\partial}{\partial\gamma}\log\sum_{g} (\Pr(z|g,t)\Pr(g|t))= \\ gE(\varepsilon|z,t)+0-\frac{\sum_{g} \frac{\partial}{\partial\gamma}(\Pr(z|g,t)\Pr(g|t))}{\sum_{g} \Pr(z|g,t)\Pr(g|t)}= \\ gE(\varepsilon|z,t)+0-\frac{\sum_{g} ((\frac{\partial}{\partial\gamma}(\Pr(z|g,t))\Pr(g|t))+\sum_{g} (\Pr(z|g,t)*(\frac{\partial}{\partial\gamma}\Pr(g|t)))}{\sum_{g} \Pr(z|g,t)\Pr(g|t)}= \\ gE(\varepsilon|z,t)+0-\frac{\sum_{g} ((\frac{\partial}{\partial\gamma}(\Pr(z|g,t))\Pr(g|t))+0}{\sum_{g} \Pr(z|g,t)\Pr(g|t)}= \\ gE(\varepsilon|z,t)+0-\frac{\sum_{g} (g*f(x))\Pr(g|t))}{\sum_{g} \Pr(z|g,t)\Pr(g|t)}= \\ \\ gE(\varepsilon|z,t)-\frac{\bar{g}f(x)}{\sum_{g} \Pr(z|g,t)\Pr(g|t)}=gE(\varepsilon|z,t)\text{ because mean(g) is 0.} \end{matrix}$$

[7] show that under the assumption of no gene environment correlation, the prospective likelihood is more powerful than the retrospective likelihood for ascertained data. In the case of the LT model, the retrospective and prospective likelihoods are the same.

Robustness to deviations in model parameters

We extended the results of Table 2 by computing the LT statistic using model parameters that were different from the values c=0.08 and m=-1.44 that we used to generate the data. Results for small deviations of c and m are displayed in Supplementary Table 1a. In each case, we find that the LT statistic is robust to small deviations in the values of these parameters. To explore large deviations of c and m, we fixed m=-1.44, effect size γ=0.1, and varied c from 0 to 0.16 (Supplementary Table 1b). At c=0 the average LT statistic is equivalent to logR and at c=0.16 the average LT statistic remains greater than logR, showing that when the prevalence is estimated correctly, even large upward changes to c result in improvement. We then fixed c=0.08, γ=0.1, and varied m from 0 to -4.0. In all cases the LT statistic outperformed LogR (Supplementary Table 1b). In summary, even when model parameters are severely mis-estimated (by up to 100%), our method still performs as well or better than LogR in all cases examined.

The LT statistic is a score test when the parameters of the model are estimated accurately (see Methods). We investigated the extreme tail of the distribution of the LT statistic under large deviations in parameters *c* and *m* to establish that mis-estimation results in a loss of power rather than an increased false positive rate. We simulated data with *c*=0.08 and *m*=-1.44 and tested the data with incorrect parameters (*c*=0.01 and *m*=-4.44) and (*c*=0.18 and *m*=-0.10). We simulated 350,000,000 studies and found that the proportion of tests with p-value < 5e-7, was 4.77e-7 and 5.17e-7 respectively.

The LT statistic assumes that there is no gene environment correlation in the population. Recently [8,9] showed that there is an inflated type-1 error rate for

some tests (including logistic regression) when this assumption is violated and that this problem is fixed by using empirical as opposed to analytical variance estimates. In the LT statistic we use the empirical variance.

Odds Ratio in the Liability Threshold Model

The liability given by *φ* = *c*(*t* – $\bar{t}$) +m+ *ε* where *ε* = *γg* + N(0,1). Assuming Hardy-Weinberg Equilibrium, for a given value of the covariate *t*, population minor allele frequency (*p*) of *g*, and effect size *γ*, the odds ratio of *g* is computed as follows: The prevalence of the disease is given by f = $\Phi(c(t-\bar{t})+m)$. The frequency of one copy of the risk allele is given by $p_{1}^{+}=\Phi(c(t-\bar{t})+m+\gamma)2p(1-p)/f$ and $p_{1}^{-}=\Phi(-c(t-\bar{t})-m+\gamma)2p(1-p)/(1-f)$ in the cases and controls respectively. The frequency of two copies of the risk allele is given by $p_{2}^{+}=\Phi(c(t-\bar{t})+m+2\gamma)p^{2}/f$ and $p_{2}^{-}=\Phi(-c(t-\bar{t})-m+2\gamma)p^{2}/(1-f)$. The frequency of no copies is given by $p_{0}^{+}=\Phi(c(t-\bar{t})+m)(1-p)^{2}/f$ and $p_{0}^{-}=\Phi(-c(t-\bar{t})-m)(1-p)^{2}/(1-f)$. The frequency in the cases is then $p^{+}=(p_{1}^{+}+2p_{2}^{+})/(2p_{2}^{+}+2p_{1}^{+}+2p_{0}^{+})$ and similarly in the controls. The allelic odds ratio is then computed in the standard way: $OR=p^{+}(1-p^{-})/(p^{-}(1-p^{+}))$. [10] provide a discussion of converting between odds ratio to liability threshold effect sizes.

We repeated the simulations from Table S1 in which m=-1.44, γ=0.1, c=0.08 and measured our LT based estimate of the odds ratio (see previous paragraph). As expected, when the LT model parameters are correctly specified, the LT odds ratio estimate (1.22) is equivalent to the standard allelic odds ratio estimate (1.22). When the LT model parameters are mis-estimated, the LT based estimate is biased. The LT based estimate was 1.20 when c=0.0 and was 1.42 when c=0.16. Note that allelic odds ratios are only equivalent to odds ratio estimates from logistic regression under certain conditions [11].

Simulations under varying ascertainment schemes

We extended the BMI-matched scheme (BMI 24 & 35) results of Table 3 to a range of different ascertainment schemes with results displayed in Supplementary Table 2(a,b,c). For Table Supplementary 2(a,b) we used a liability threshold model with parameters c = 0.08 and m = -1.44 just as in Table 2, with a minor allele frequency of 0.5 in the populations and an effect size *γ* of 0.1.

We tested a case-control ascertainment study with randomly ascertained BMI (Case-Control Random BMI) by generating individuals under the LT model and randomly assigning a BMI=24 or BMI=35 until we had 3,000 cases and 3,000 controls. Supplementary Table 2(a) shows that average BMI is much larger in cases than in controls. LT outperforms LogR in this case.

We tested a case-control-covariated study with low-BMI cases (Case-Control Low BMI), similar to the T2D Metabochip study, by generating individuals as we did for Table 2 except that there were 2500 cases with BMI=24 and 500 cases with BMI=35. In this case both LogR and LT are greater than they were in Table 2 for the same effect size, and LT still outperforms LogR.

We tested a cross-sectional study (Cross-Sectional) by randomly generating 10,000 individuals under the same LT model. On average there were 1,500 cases and 8,500 controls with the average case/control ages matching those of the Case-Control Random BMI study. In this case the probit regression statistic with BMI covariate is similar to the LT statistic and they have the same average statistic. The LT statistic is given the effect size of BMI and so there exists the theoretical possibility that it will outperform probit regression, which must estimate the effect size from the observed data. In practice, given typical GWAS sample sizes, the effect size of strong clinical covariates are easily estimated from the data.

Ratio of χ^2^ as a function of covariate effect size and disease prevalence. We examined the improvement of the LT relative to the LogR and LogR+Cov statistics as a function of the disease prevalence and covariate effect size. For a given prevalence and fraction of variance explained by the covariate we simulated 1,000 case-control ascertainment studies under a liability threshold model with a SNP minor allele frequency of 20% and an effect size on the liability scale of 0.1. The results are displayed in Supplementary Table 3.

The improvement of the LT relative to the LogR and LogR+Cov increases with covariate effect size. The effect of prevalence is a function of the test statistic. When the disease prevalence is small, the improvement of LT over LogR is greater than the improvement of LT over LogR+Cov. When the disease prevalence is high, the improvement of LT over LogR+Cov is greater than the improvement of LT over LogR.

Non-Additive Models

We tested a model in the same simulation framework as above with gene x BMI interaction under the liability scale by adding a positive interaction term of 0.05*g*BMI where g and BMI were normalized to have mean 0 and variance 1 (GxE) and similarly for a negative interaction term -0.05*g*BMI (-GxE). The results are shown in Supplementary Table 4.

In the GxE case the LT statistic underperformed and the G+GxE statistic outperformed the other models since it is the only one explicitly modeling this type of interaction. The LT is expected to underperform other models because the interaction term will generate an effect opposite to that predicted by the model. Without interaction the LT model requires more risk alleles to exceed the liability. With interaction, the frequency of both the risk allele and high BMI individuals are increased in cases, reducing the LT statistic.

In the –GxE case the LT statistic and the G+GxE statistic both outperformed the other models, with LT outperforming G+GxE. In this case, the interaction term exaggerates the prediction of the LT model (i.e. cases with low BMI will have more copies of the risk allele), which thus outperforms other tests. Averaging across the –GxE and GxE scenarios, LT outperforms both LinR and LogR and we therefore recommend LT over these tests even in the presence of gene by environment interactions.

Simulations with Real Covariate Data

We simulated data as described for Supplementary Table 2, but using the case-control status and covariates taken from real data sets. For example, we took the real age and case-control status data from all individuals in the prostate cancer data set and used the LTPub model with a minor allele frequency of 0.05 and an effect size *γ* of 0.07. For a case individual, we drew random genotypes and *ε* until a *φ*>0 was achieved and similarly for controls until *φ*<0. Given this set of genotypes, we applied all of the set of statistical tests and repeated this entire process 100,000. We computed the average ratio and variance of LT to LogR statistics. The sample sizes here are the same as those in the real studies and missing data covariate data was replaced with the appropriate average in cases or controls. The results are shown in Supplementary Table 5. The LTPub model parameters were based on optimizing the squared error on the disease scale and were nearly identical to those estimated on the liability scale.

Permutation Tests for Real Data Sets

We performed permutation tests to determine if the improvement of the LT relative to LogR was a function of leveraging the covariates effectively rather than a consequence of differences in the distributions of covariates between cases and controls. For each data set we permuted the covariates within cases and controls separately and recomputed the sum of LT statistics 100,000 times. We counted the fraction of times a permuted data set had a sum of LT statistics exceeding that of the un-permuted data and found that for type 2 diabetes, lung cancer, prostate cancer, and the ESKD data sets, this occurred zero times (P < 10^-5^). In the age-related macular degeneration, breast cancer, and rheumatoid arthritis data sets this occurred less than 300 times (P < 0.03). If the gains in LT were solely a function of the difference in the covariate between cases and controls then permuting within cases and controls would not have produced significant results.

Estimating LT parameters by optimizing over known associated variants (LTFit). In order to investigate the robustness of the LTPub parameter estimation approach that we recommend as the method of choice, we compared LTPub to a completely different parameter estimation approach, LTFit. Given sufficiently many known variants, LTFit learns the LT parameters directly from the data. The method proceeds via an empirical Bayes approach as follows: For a given parameter choice we compute the sum of χ^2^ statistics over all known associated variants in the data. We then follow a coarse grid search to find the parameter choice that maximizes the χ^2^ statistics of those SNPs. We choose a coarse grid since small changes in the parameters have little effect of the test statistic (see Supplementary Table 1). We caution that if there are less than 15 known associated variants with χ^2^ > 5 this method may result in over fitting. Unlike the LTPub method above, the LTFit method does not require the independence assumption for multiple covariates such as age and BMI in the T2D study. However, the robustness of the LTFit method will depend on the number of samples, number of known associated variants, and strength of associations; for this reason we do not recommend the broad application of the LTFit method.

We used a leave-one-out procedure in our real data sets to ensure that LTFit parameters were estimated in out-of-sample fashion, in the sense that the candidate SNP was never included in the set of SNPs used to estimate LTFit parameters that were used to compute LT association statistics for the candidate SNP. This prevents overestimates of the benefits of the LTFit model. We excluded each SNP in turn and performed the grid search described above on all other SNPs. Thus if there are 19 SNPs in the data set, we estimated LTFit parameters 19 times. In our data sets the optimal parameters on the grid were identical for all SNPs in all data sets tested with LTFit.

For the T2D, prostate cancer, post-menopausal breast cancer, and rheumatoid arthritis data sets, there were sufficiently many known associated variants to estimate parameters by optimizing over known associated variants (LTFit). For T2D the parameters estimated from this method were slightly larger than the ones estimate from the literature and there were slight gains in power. However, we continue to recommend LTPub parameters for T2D. We note that the smaller LTPub parameter values are more conservative, since LT is equivalent to ATT when c parameters are equal to 0. For the other diseases, the parameters estimated by LTFit matched the LTPub estimates (Supplementary Table 7). The values of the LT statistics for each SNP computed from the LTFit model parameters are given in Supplementary Tables 8-15. From a robustness standpoint it is reassuring that there exists an independent parameter estimation method (LTFit) that produces similar results to the recommended approach (LTPub) for the subset of diseases to which LTFit can be applied.

Genetically Influenced Covariates. Some clinical covariates, such as age are not influenced by genetic factors and can be included it the LT model without concern of confounding due to shared genetics with the phenotype of interest. Other clinical covariates such as BMI and smoking addiction maybe related to disease status (e.g. T2D and lung cancer) and have a genetic basis themselves. In this case, including the covariate may alter the power to identify SNPs associated with phenotype via the covariate. Consider a SNP that alters the BMI of an individual, which in turn alters T2D risk. In the case of standard linear or logistic regression, the effect of BMI is conditioned out and so the SNP will not be discovered in the analysis. Under the LT statistic there will be a reduced association between the SNP and the posterior mean of the residual of the liability. However, unlike the linear or logistic regression case, the LTSCORE may still generate a significant test statistic for a SNP that modulates T2D risk via BMI. It is straightforward to test for this possibility by performing logistic regression conditioned on BMI for all SNPs found to be significant under the LT statistic.

Simulations under the logit model. We examine the use of the logit model as the generating model of disease as opposed to the liability threshold model (probit model) used in the other simulations described above. Under a logit model the probability that an individual is a case is $P(z=1)=\frac{1}{e^{c(t-\bar{t})+\gamma g+m}+1}$, where $\gamma$ is the genotype effect size, c is the clinical covariate effect size, and m is an affine term that determines the disease prevalence. We used random draws from a binomial distribution with minor allele frequency 20% for the genotype and a normally distributed random variable with mean 0 and variance 1 for the clinical covariate. We generated case-control studies with 1,000 cases and 1,000 controls and examined the performance of LT and logistic regression with and without a covariate for a range of prevalences and effect sizes.

In practice, the LT parameters are estimated from epidemiological data and in order to be maximally conservative we simulated epidemiological scale data to estimate the LT parameters. We randomly generated 60,000 individuals under the same logit model used for the simulation. We split the 60,000 individuals into six subgroups and measured the mean of the clinical covariate and the prevalence in that subgroup. We then estimated the LT parameters (see Methods) and used these estimated parameters when computing the LT statistics. We assume that the prevalence data collected from the epidemiological literature and the covariates measured in the study are reasonably accurate. If there are large errors in either of the measurements it will cause a drop in the power of our method.

The results shown in Table 6 demonstrate that the LT statistic outperforms the other tests even when the data are generated under the logit model and the parameters are estimated from summary epidemiological data collected from 60,000 individuals. This shows that the gain in power is not a function of having the correct model, but rather a function of leveraging the external epidemiological data to model disease prevalence.

Frequency of risk alleles in cases. We simulated a 1,000,000 case control studies of 1,000 cases and 1,000 under a liability threshold model with $\gamma$= 0.2, c=0.5, and m=1.65. We used the sum of two random binomial variables with minor allele frequency 20% to represent the genotype and a random binomial with frequency 50% to represent the environmental covariate. We measured the frequency of the risk allele in low-risk (t=0) and high-risk (t=1) individuals and found the frequencies were 90% and 82% respectively. We repeated this analysis using a logit model with $\gamma$= 0.405, c=1.5, and m=4 and found that the frequencies were 67% and 64% for the low and high-risk cases respectively. We conclude that under either a logit or probit model of disease, there is an increase in risk minor allele frequency for low-risk cases relative to high-risk cases. Under either model, individuals in the high-risk category do not need as much help from genetic variants to achieve a fixed probability of being a case. This corresponds to an overall lower average risk minor allele frequency in high-risk cases. There may exist additional disease models in which this is not the case and in such situations, the LT statistic may not outperform logistic regression.

Comparison with Chatterjee and Carroll prospective likelihood. We repeated the experiments of the previous section and applied the prospective likelihood approach described in[7]. The results shown in Table 6 demonstrate that the LT statistic outperforms this semi-parametric approach even when the case-control ascertainment data are generated under a logit model. Furthermore, the LT statistic will increase power when the study uses a balanced design (e.g. age-matched design) while the Chatterjee and Carroll method, like other conditioning approaches, will not improve upon logistic regression with no covariate. This suggests that novel loci may be identified by applying our method to existing and future association studies of these diseases.

29 Recent Studies. We compiled a list of 29 recent studies, all published in the journal *Nature Genetics*, which either ignored non-genetic covariates or applied standard conditioning approaches. We believe there is a large potential for the LT method described here to identify new variants in these studies [12,13,14,15,16,17,18,19,20,21,22,23,24,25,26,27,28,29,30,31,32,33,34,35,36,37,38,39,40].

Additional approaches to analysis of case-control ascertained data. In addition to the methods described in the main text for analysis of case-control ascertained data[41] describe a method that splits cases into low-risk and high-risk groups to account for covariate heterogeneity or GxE interaction in case-control ascertained association studies. [42,43] propose methods such as inverse-probability weighting to account for case-control ascertainment issues when conditioning on clinical covariates. Although the LT statistic outperformed inverse-probability weighting in terms of increasing study power, inverse-probability weighting remains a valuable approach in the alternate context of avoiding confounding in association tests with secondary traits. [44] proposes a two-stage design to identify GxE interactions. While these methods share some similarities with the LT method they do not model risk as a continuous function of clinical covariates, estimate parameters based on known prevalence information or associated variants, handle multiple covariates, and most importantly, they do not handle the case-control-covariate case.

Additional covariates. In addition to the clinical covariates, such as BMI and age, used to increase power via the LT method, researchers may wish to include additional covariates such as principal components[45] for the purpose of preventing false positives. While there may exist epidemiological data relating these covariates to prevalence data, including them in the computation of posterior mean liabilities (stage-1) will not prevent false-positives. However, including them as standard covariates in the linear regression stage of our approach (stage-3) will have the desired effect of preventing false-positives[46].

Researchers can also include covariates for the purposes of refocusing studies, for example, away from known pathways. Consider the example of a researcher who wishes to discover genetic variation related to lung-cancer that does not act via smoking related pathways. In such cases, smoking status (or other pathway-blocking covariates) must be included as a fixed effect in the linear regression stage (stage-3) of the LT method. If a SNP is not associated when conditioned on smoking status under logistic regression, then it will not be significant under the LT statistic when including smoking status as a fixed effect. The reasoning is similar to that of conditioning on principal components. See [46] for a detailed explanation.

Supplementary Tables

Supplementary Table 1. Average χ^2^ statistics for LT using incorrect model parameters. We display average results across 1,000,000 simulations for the LT statistic using various values of the parameter *c* and *m* as compared to the values c=0.08 and m=-1.44 used to generate the data. The LogR and LT columns of the table correspond to Table 1. We further note that the LT statistic using *c*=0.00 is roughly equivalent to LogR. All statistics had the correct null distribution at γ=0. Table (a) shows modest deviations of c,m over a range of γ. Table (b) shows large deviations of of c,m for γ=0.1.

| *γ* | LogR | LT | LT, *c*=0.07 and *m*=-1.30 | LT, *c*=0.09 and *m*=-1.67 | LT, *c*=0.08 and *m*=-1.08 | LT, *c*=0.08 and *m*=-1.88 |
| --- | --- | --- | --- | --- | --- | --- |
| 0.10 | 27.88 | 30.34 | 30.32 | 30.32 | 30.34 | 30.34 |
| 0.11 | 33.45 | 36.48 | 36.55 | 36.55 | 36.53 | 36.48 |
| 0.12 | 39.77 | 43.46 | 43.57 | 43.57 | 43.56 | 43.46 |
| 0.13 | 45.92 | 50.29 | 50.43 | 50.42 | 50.43 | 50.30 |
| 0.14 | 52.74 | 57.79 | 57.84 | 57.82 | 57.81 | 57.79 |
| 0.15 | 59.63 | 65.55 | 65.66 | 65.66 | 65.65 | 65.54 |

(a)

| γ | LogR | LT, c=0.0  m=-1.44 | LT,  c=0.02  m=-1.44 | LT,  c=0.4  m=-1.44 | LT, c=0.10 m=-1.44 | LT, c=0.12  m=-1.44 | LT, c=0.16  m=-1.44 |
| --- | --- | --- | --- | --- | --- | --- | --- |
| 0.10 | 27.88 | 27.89 | 28.84 | 30.01 | 30.30 | 30.19 | 29.83 |
| γ | LogR | LT, c=0.08  m=0 | LT,  c=0.08  m=-0.2 | LT, c=0.08  m=-0.7 | LT, c=0.08  m=-2.0 | LT, c=0.08  m=-3.0 | LT, c=0.08  m=-4.0 |
| 0.10 | 27.88 | 29.57 | 30.17 | 30.23 | 30.09 | 29.56 | 29.19 |

(b)

Supplementary Table 2. Average χ^2^ statistics for LT vs. other approaches under varying ascertainment schemes. (a) We display the average test statistic across 100,000 simulations for various study designs. (b) We display the power for different test statistics across 100,000 simulations for various study designs.

(a)

| Study Design | Average  Case BMI | Average Control BMI | LogR | LogR+  Cov | GxE | G+GxE | LT |
| --- | --- | --- | --- | --- | --- | --- | --- |
| Case Control Random BMI | 32.89 | 28.90 | 22.00 | 22.74 | 1.27 | 20.64 | 23.65 |
| Case Control  Low BMI | 25.83 | 33.17 | 38.47 | 16.16 | 1.19 | 14.31 | 40.16 |
| Cross-Sectional  Random BMI | 32.89 | 28.90 | 18.84 | 20.11 | 1.19 | 18.06 | 20.40 |

(b)

| Study Design | LogR | LogR+cov | GxE | G+GxE | LT |
| --- | --- | --- | --- | --- | --- |
| Case Control Random BMI | 0.19 | 0.21 | 0.00 | 0.16 | 0.25 |
| Case Control  Low BMI | 0.75 | 0.06 | 0.00 | 0.04 | 0.79 |
| Cross-Sectional | 0.11 | 0.14 | 0.00 | 0.10 | 0.15 |

Supplementary Table 3. Ratio of χ^2^ as a function of covariate effect size and disease prevalence. The average ratio of χ^2^ statistics as a function of disease prevalence (rows) and fraction of variance explained by the covariate (columns) on the liability scale. We used a SNP with a minor allele frequency of 20%, an effect size of 0.1, and generated case control data 1000 times for each effect size and prevalence. Table (a) is the average ratio of LT to LogR and Table (b) is the average ratio of LT to LogR+Cov.

(a)

|  | 0.10% | 0.50% | 1% | 5% | 10% |
| --- | --- | --- | --- | --- | --- |
| 1% | 1.00 | 1.00 | 1.00 | 1.00 | 1.00 |
| 5% | 1.01 | 1.01 | 1.01 | 1.01 | 1.01 |
| 10% | 1.01 | 1.01 | 1.02 | 1.02 | 1.03 |
| 20% | 1.03 | 1.04 | 1.04 | 1.07 | 1.07 |
| 50% | 1.12 | 1.13 | 1.18 | 1.16 | 1.26 |

(b)

|  | 0.10% | 0.50% | 1% | 5% | 10% |
| --- | --- | --- | --- | --- | --- |
| 1% | 1.02 | 1.02 | 1.01 | 1.00 | 1.00 |
| 5% | 1.07 | 1.05 | 1.04 | 1.00 | 1.00 |
| 10% | 1.16 | 1.10 | 1.06 | 1.02 | 1.00 |
| 20% | 1.27 | 1.16 | 1.14 | 1.03 | 1.02 |
| 50% | 1.41 | 1.27 | 1.21 | 1.12 | 1.04 |

Supplementary Table 4. Interaction Models. Average test statistics (a) and power (b) for non-additive models.

(a)

| Interaction | Average  Case BMI | Average Control BMI | LogR | LogR+  Cov | GxE | G+GxE | LT |
| --- | --- | --- | --- | --- | --- | --- | --- |
| -GxE | 29.50 | 29.50 | 31.74 | 31.84 | 18.79 | 42.40 | 47.55 |
| GxE | 29.50 | 29.50 | 24.11 | 24.12 | 10.63 | 31.00 | 16.90 |

(b)

| Interaction | LogR | LogR+Cov | GxE | G+GxE | LT |
| --- | --- | --- | --- | --- | --- |
| -GxE | 0.54 | 0.54 | 0.11 | 0.83 | 0.91 |
| GxE | 0.26 | 0.26 | 0.01 | 0.51 | 0.07 |

Supplementary Table 5. Simulations with Real Covariate Data. We display the average test statistic across 100,000 simulations using the real covariates and case-control status in the breast cancer (BC), rheumatoid arthritis (RA), prostate cancer (PC), and type 2 diabetes (T2D) data sets. Ratio average is the average ratio of LT to LogR across the 100,000 simulations and Ratio Std Dev, is the standard deviation of the ratio.

| Study Design | ***γ*** | maf | LogR | LogR+Cov | LT | Ratio Average | Ratio Std Dev |
| --- | --- | --- | --- | --- | --- | --- | --- |
| BC | 0.07 | 0.05 | 15.54 | 15.67 | 16.18 | 1.05 | 0.02 |
| RA | 0.07 | 0.05 | 9.39 | 9.49 | 9.67 | 1.03 | 0.01 |
| PC | 0.07 | 0.05 | 12.62 | 12.91 | 13.18 | 1.06 | 0.03 |
| T2D Mec | 0.10 | 0.10 | 76.44 | 84.52 | 92.83 | 1.22 | 0.06 |
| T2D Metabo | 0.10 | 0.10 | 12.57 | 14.39 | 16.30 | 1.40 | 0.04 |

Supplementary Table 6. Average χ^2^ statistics for LT vs. other approaches under logit generated data. We display the average test statistic across 100,000 simulations for various study designs. OR = odds ratio. Prev = Prevalence.

| Study Design | LogR | LogR+  Cov | LT | Chaterjee  Caroll |
| --- | --- | --- | --- | --- |
| OR=1.2  Prev = 4.4% | 14.16 | 12.47 | **15.33** | 14.26 |
| OR = 1.3  Prev = 4.5% | 28.61 | 25.22 | **31.53** | 28.98 |
| OR = 1.4  Prev = 4.6% | 46.28 | 41.23 | **52.10** | 47.17 |

Supplementary Table 6. Summary statistics for all data sets. Sum of statistics of the tests covered in the main text as well as probit regression, and the GxE test of interaction. In all cases, sum χ^2^ for LT exceeds other tests.

| Disease | LT | LogR | LogR+Cov | GxE | G+GxE |
| --- | --- | --- | --- | --- | --- |
| T2D (Metabo) | 369.7 | 244.05 | 252.23 | 66.37 | 228.43 |
| T2D (MEC) | 402.86 | 320.08 | 400.89 | 51.98 | 396.34 |
| PC | 1912.88 | 1787.61 | 1844.40 | 44.61 | 1743.96 |
| LC | 416.95 | 359.64 | 331.28 | 32.54 | 321.12 |
| BC | 395.16 | 390.86 | 386.83 | 14.53 | 353.63 |
| RA | 511.31 | 470.91 | 466.11 | 37.58 | 465.68 |
| ESKD | 188.38 | 137.80 | 134.70 | 8.50 | 163.64 |
| AMD | 185.6 | 159.38 | 110.33 | 3.30 | 134.83 |

Supplementary Table 7. LTFit and LTPub Models. The LT model parameters found by LTPub and LTFit for the diseases with sufficiently many independent SNPs to run LTFit.

| Disease | *φ* (LTPub) | *φ* (LTFit) |
| --- | --- | --- |
| T2D (Metabo) | 0.08*(bmi-26.5)+0.029*(age-50)-1.4 | 0.115*(bmi-26.5)+0.034*(age-50)-1.64 |
| T2D (MEC) | 0.08*(bmi-26.5)+0.029*(age-50)-1.4 | 0.15*(bmi-26.5)+0.046*(age-50)-1.45 |
| PC | 0.049*(age-50)-2.49 | 0.06*(age-50)-2.4 |
| BC | 0.032*(age-50)-2.26 | 0.032*(age-50)-2.26 |
| RA | 0.022*(age-50)+0.32*(sex-0.5)-2.46 | 0.024*(age-50)+0.34*(sex-0.5)-2.58 |

Supplementary Table 8. χ^2^ statistics for LT vs. other approaches in the T2D Metabochip data set. OR LBMI is the odds ratio computed from cases with BMI less than the median case BMI. OR HBMI is the odds ratio for cases with BMI greater than median BMI. *Indicates the SNP is in the BMI associated FTO locus.

| rsid | LTPub | LTFit | LogR | LogR+Cov | OR LBMI | OR HBMI |
| --- | --- | --- | --- | --- | --- | --- |
| chr1:120319482 | 8.37 | 8.72 | 3.48 | 3.47 | 1.13 | 1.09 |
| rs340874 | 2.00 | 2.10 | 0.82 | 0.79 | 1.11 | 1.07 |
| rs780094 | 0.00 | 0.01 | 0.04 | 0.00 | 1.07 | 1.08 |
| rs11899863 | 8.11 | 8.87 | 4.66 | 4.62 | 1.21 | 1.15 |
| rs243083 | 0.71 | 0.51 | 0.52 | 0.13 | 1.07 | 1.11 |
| rs2925757 | 1.04 | 1.34 | 0.08 | 0.00 | 1.05 | 0.99 |
| rs7578326 | 3.58 | 4.06 | 1.34 | 0.97 | 1.14 | 1.04 |
| chr3:12368125 | 6.99 | 6.75 | 4.78 | 4.72 | 1.14 | 1.13 |
| rs6795735 | 4.71 | 3.77 | 6.32 | 6.35 | 1.10 | 1.12 |
| chr3:64686944 | 1.07 | 0.82 | 0.99 | 0.96 | 1.08 | 1.11 |
| rs11708067 | 6.28 | 7.19 | 1.82 | 1.80 | 1.11 | 1.05 |
| rs4402960 | 6.65 | 8.13 | 1.79 | 2.42 | 1.20 | 1.17 |
| chr4:6343816 | 8.44 | 7.92 | 5.27 | 5.65 | 1.14 | 1.13 |
| chr4:6353923 | 8.23 | 7.84 | 5.21 | 5.52 | 1.14 | 1.13 |
| rs4457053 | 9.07 | 9.74 | 3.30 | 2.70 | 1.15 | 1.09 |
| chr6:20769229 | 5.44 | 4.80 | 5.00 | 5.09 | 1.06 | 1.09 |
| rs9368222 | 9.68 | 9.11 | 7.20 | 7.40 | 1.11 | 1.14 |
| rs2191349 | 5.53 | 6.65 | 0.87 | 0.98 | 1.10 | 1.07 |
| chr7:28147081 | 3.46 | 3.59 | 1.27 | 1.36 | 1.11 | 1.10 |
| chr7:44202193 | 0.61 | 0.52 | 1.15 | 1.03 | 1.15 | 1.12 |
| chr7:130117394 | 0.38 | 0.27 | 0.43 | 0.37 | 1.04 | 1.02 |
| rs896854 | 8.87 | 8.70 | 6.64 | 6.94 | 1.15 | 1.07 |
| rs13266634 | 17.40 | 17.39 | 13.46 | 15.01 | 1.20 | 1.09 |
| rs3802177 | 17.21 | 17.19 | 13.25 | 14.77 | 1.19 | 1.09 |
| rs2383208 | 12.24 | 11.19 | 10.58 | 10.56 | 1.24 | 1.17 |
| rs10965250 | 16.19 | 14.96 | 13.59 | 13.02 | 1.28 | 1.20 |
| rs17791513 | 10.27 | 8.57 | 12.51 | 11.08 | 1.22 | 1.15 |
| rs7069060 | 1.65 | 2.03 | 0.52 | 0.74 | 1.05 | 1.01 |
| rs1111875 | 3.03 | 3.22 | 1.96 | 2.43 | 1.14 | 1.12 |
| rs7903146 | 94.58 | 103.99 | 50.46 | 54.90 | 1.69 | 1.30 |
| rs2334499 | 5.73 | 6.01 | 3.01 | 3.12 | 1.05 | 1.01 |
| rs231362 | 5.00 | 4.98 | 3.73 | 3.78 | 1.09 | 1.05 |
| rs163184 | 11.82 | 11.75 | 6.85 | 5.81 | 1.09 | 1.08 |
| chr11:2813770 | 5.48 | 6.29 | 1.04 | 0.73 | 1.10 | 1.06 |
| rs2237897 | 3.22 | 2.70 | 1.28 | 1.13 | 1.29 | 1.31 |
| rs5215 | 7.20 | 7.14 | 5.73 | 7.00 | 1.10 | 1.05 |
| rs5219 | 6.97 | 6.93 | 5.48 | 6.78 | 1.09 | 1.05 |
| rs1552224 | 7.50 | 8.28 | 3.43 | 4.44 | 1.14 | 1.06 |
| rs1387153 | 5.63 | 5.20 | 4.26 | 4.32 | 1.05 | 1.04 |
| rs2261181 | 0.92 | 0.63 | 1.07 | 0.97 | 1.13 | 1.14 |
| rs7138300 | 13.07 | 13.26 | 11.89 | 12.97 | 1.13 | 1.07 |
| chr12:119945069 | 5.11 | 4.39 | 4.39 | 3.75 | 1.13 | 1.09 |
| rs11634397 | 0.01 | 0.11 | 0.12 | 0.00 | 1.03 | 0.96 |
| rs12899811 | 5.88 | 5.13 | 5.94 | 5.86 | 1.15 | 1.11 |
| rs8050136* | 0.00 | 0.15 | 0.66 | 0.22 | 1.08 | 1.19 |
| rs11642841* | 0.95 | 0.47 | 2.31 | 1.64 | 1.10 | 1.13 |
| rs7501939 | 3.43 | 3.09 | 3.53 | 3.90 | 1.16 | 1.13 |
| SUM | 369.70 | 376.47 | 244.05 | 252.23 | n/a | n/a |

Supplementary Table 9. χ^2^ statistics for LT vs. other approaches in the T2D MEC data set. OR LBMI is the odds ratio computed from cases with BMI less than the median case BMI. OR HBMI is the odds ratio for cases with BMI greater than median BMI. *Indicates the SNP is in the BMI associated FTO locus.

| rsid | LTPub | LTFit | LogR | LogR+Cov | OR LBMI | OR HBMI |
| --- | --- | --- | --- | --- | --- | --- |
| rs4430796 | 4.18 | 4.44 | 3.57 | 4.12 | 1.02 | 0.96 |
| rs8050136* | 0.68 | 0.12 | 3.93 | 0.03 | 1.15 | 0.89 |
| rs10010131 | 14.01 | 14.14 | 11.75 | 12.25 | 1.49 | 1.01 |
| rs10923931 | 1.21 | 0.87 | 2.27 | 1.05 | 1.19 | 0.89 |
| rs1111875 | 6.23 | 6.61 | 4.56 | 5.73 | 1.06 | 0.88 |
| rs12779790 | 7.06 | 7.56 | 5.19 | 7.67 | 1.10 | 1.07 |
| rs13266634 | 21.88 | 22.81 | 16.57 | 19.50 | 1.05 | 1.16 |
| rs1801282 | 6.09 | 7.06 | 3.94 | 7.84 | 1.20 | 1.02 |
| rs2237895 | 39.58 | 42.85 | 25.75 | 38.45 | 1.25 | 1.10 |
| rs2237897 | 54.73 | 57.70 | 38.73 | 52.02 | 1.01 | 0.85 |
| rs2383208 | 30.62 | 30.96 | 25.07 | 29.56 | 1.01 | 1.23 |
| rs4402960 | 18.80 | 20.55 | 12.94 | 18.49 | 1.08 | 1.06 |
| rs4607103 | 2.45 | 2.39 | 2.24 | 1.81 | 1.05 | 0.95 |
| rs5219 | 20.48 | 20.93 | 17.61 | 22.50 | 1.25 | 1.07 |
| rs7578597 | 6.90 | 7.86 | 4.37 | 8.51 | 1.56 | 0.98 |
| rs7754840 | 45.72 | 45.28 | 40.02 | 41.68 | 1.11 | 1.14 |
| rs7903146 | 83.38 | 84.03 | 71.72 | 86.00 | 1.13 | 1.29 |
| rs7961581 | 0.00 | 0.00 | 0.06 | 0.06 | 1.08 | 1.03 |
| rs864745 | 38.87 | 42.26 | 29.78 | 43.61 | 1.31 | 1.09 |
| SUM | 402.86 | 418.42 | 320.08 | 400.89 | n/a | n/a |

Supplementary Table 10. χ^2^ statistics for LT vs. other approaches in prostate cancer data set. OR Young is the odds ratio computed from cases with age less than the median case age. OR Old is the odds ratio computed from cases with age greater than median age. The OR results matched Lindstrom et al[47] when age=65 was used to define Old and Young.

| rsid | LTPub | LTFit | LogR | LogR+Cov | OR Young | OR Old |
| --- | --- | --- | --- | --- | --- | --- |
| rs721048 | 20.28 | 20.79 | 19.77 | 18.97 | 1.14 | 1.07 |
| rs1465618 | 19.77 | 19.79 | 19.58 | 18.78 | 1.11 | 1.10 |
| rs12621278 | 10.07 | 9.70 | 10.40 | 10.63 | 1.16 | 1.16 |
| rs2660753 | 16.30 | 16.24 | 15.64 | 15.55 | 1.14 | 1.15 |
| rs4857841 | 29.80 | 29.25 | 31.34 | 31.15 | 1.12 | 1.14 |
| rs17021918 | 20.23 | 21.07 | 17.20 | 16.36 | 1.12 | 1.08 |
| rs12500426 | 10.85 | 11.17 | 9.98 | 9.52 | 1.08 | 1.07 |
| rs7679673 | 38.82 | 39.97 | 37.88 | 35.00 | 1.16 | 1.08 |
| rs9364554 | 12.39 | 12.82 | 10.56 | 10.05 | 1.11 | 1.04 |
| rs10486567 | 55.84 | 54.93 | 56.83 | 56.11 | 1.18 | 1.20 |
| rs6465657 | 29.07 | 29.42 | 27.15 | 26.03 | 1.12 | 1.10 |
| rs1512268 | 28.01 | 28.57 | 25.57 | 24.29 | 1.15 | 1.07 |
| rs2928679 | 9.23 | 9.19 | 9.29 | 9.12 | 1.06 | 1.06 |
| rs4961199 | 6.18 | 5.89 | 6.32 | 6.32 | 1.05 | 1.09 |
| rs1016343 | 84.73 | 85.61 | 82.68 | 79.46 | 1.30 | 1.20 |
| rs7841060 | 81.20 | 82.46 | 78.32 | 74.88 | 1.30 | 1.18 |
| rs16901979 | 45.45 | 44.25 | 45.55 | 44.96 | 1.50 | 1.41 |
| rs620861 | 47.19 | 48.08 | 43.82 | 41.95 | 1.22 | 1.10 |
| rs6983267 | 120.96 | 121.98 | 114.77 | 111.16 | 1.31 | 1.16 |
| rs1447295 | 115.78 | 118.09 | 110.01 | 104.68 | 1.51 | 1.27 |
| rs4242382 | 126.75 | 129.33 | 124.37 | 115.29 | 1.51 | 1.28 |
| rs7837688 | 101.25 | 104.06 | 95.14 | 89.87 | 1.47 | 1.24 |
| rs16902094 | 32.89 | 33.33 | 29.48 | 29.23 | 1.19 | 1.16 |
| rs1571801 | 6.96 | 6.59 | 7.28 | 7.42 | 1.06 | 1.06 |
| rs10993994 | 107.03 | 108.05 | 105.59 | 100.43 | 1.25 | 1.19 |
| rs4962416 | 16.30 | 16.53 | 15.79 | 15.02 | 1.09 | 1.08 |
| rs7127900 | 30.11 | 30.07 | 28.49 | 28.00 | 1.17 | 1.12 |
| rs12418451 | 28.87 | 28.71 | 29.01 | 28.92 | 1.12 | 1.14 |
| rs7931342 | 69.29 | 67.05 | 71.20 | 73.15 | 1.19 | 1.21 |
| rs10896449 | 82.37 | 80.01 | 83.41 | 85.58 | 1.21 | 1.22 |
| rs11649743 | 29.78 | 31.26 | 28.79 | 25.07 | 1.18 | 1.12 |
| rs4430796 | 125.25 | 127.47 | 124.12 | 117.66 | 1.28 | 1.22 |
| rs7501939 | 88.39 | 89.43 | 86.52 | 83.76 | 1.23 | 1.21 |
| rs1859962 | 82.33 | 84.23 | 78.24 | 73.23 | 1.23 | 1.14 |
| rs266849 | 7.60 | 7.73 | 7.32 | 6.93 | 1.11 | 1.04 |
| rs2735839 | 22.56 | 23.24 | 21.39 | 19.63 | 1.18 | 1.10 |
| rs5759167 | 53.50 | 53.63 | 51.03 | 49.70 | 1.19 | 1.13 |
| rs5945572 | 44.86 | 44.86 | 43.88 | 43.30 | 1.18 | 1.14 |
| rs5945619 | 54.64 | 55.47 | 52.73 | 50.46 | 1.20 | 1.14 |
| SUM | 1912.88 | 1930.33 | 1787.61 | 1844.40 | n/a | n/a |

Supplementary Table 11. χ^2^ statistics for Lung Cancer data set. OR Young is the odds ratio computed from cases with age less than the median case age. OR Old is the odds ratio for cases with age greater than median age. LTFit was not included because the number of independent SNPs was too low. *Indicates SNPs removed when using both age and smoking as covariates.

| Rsid | LT | LogR | LogR+Cov | OR Young | OR Old |
| --- | --- | --- | --- | --- | --- |
| rs1051730* | 124.36 | 106.01 | 96.25 | 1.30 | 1.22 |
| rs12050604* | 8.86 | 8.73 | 7.30 | 0.92 | 1.32 |
| rs1530057* | 0.85 | 0.40 | 0.00 | 1.03 | 1.54 |
| rs1926203* | 0.01 | 0.00 | 0.44 | 1.00 | 1.11 |
| rs2352028* | 4.08 | 3.94 | 1.68 | 0.95 | 1.25 |
| rs2736100 | 13.45 | 10.31 | 12.67 | 0.92 | 1.11 |
| rs2808630* | 2.11 | 2.88 | 0.14 | 0.95 | 1.00 |
| rs3117582 | 7.54 | 13.40 | 15.24 | 1.17 | 1.09 |
| rs401681 | 16.95 | 13.03 | 14.40 | 1.09 | 1.43 |
| rs402710 | 45.76 | 35.77 | 31.64 | 1.15 | 1.44 |
| rs4254535* | 0.05 | 0.00 | 0.01 | 1.00 | 1.15 |
| rs4975616 | 17.19 | 12.91 | 12.84 | 1.09 | 1.51 |
| rs748404* | 7.57 | 9.54 | 2.85 | 0.91 | 1.06 |
| rs7626795* | 1.92 | 2.56 | 0.61 | 0.94 | 1.17 |
| rs8034191* | 102.63 | 84.06 | 74.69 | 1.26 | 1.13 |
| rs8042374* | 63.64 | 56.09 | 60.52 | 1.25 | 1.91 |
| SUM | 416.95 | 359.64 | 331.28 | n/a | n/a |

Supplementary Table 12. χ^2^ statistics for Breast Cancer data set. OR Young is the odds ratio computed from cases with age less than the median case age. OR Old is the odds ratio for cases with age greater than median age.

| Rsid | LTPub | LTFit | LogR | LogR+Cov | OR Young | OR Old |
| --- | --- | --- | --- | --- | --- | --- |
| rs889312 | 21.71 | 21.72 | 21.58 | 18.80 | 1.11 | 1.10 |
| rs1045485 | 8.92 | 8.92 | 9.25 | 7.47 | 1.09 | 1.12 |
| rs1799930 | 0.00 | 0.00 | 0.03 | 0.00 | 1.00 | 0.99 |
| rs1799931 | 0.47 | 0.47 | 0.31 | 0.15 | 1.10 | 1.01 |
| rs1801280 | 0.48 | 0.48 | 0.46 | 0.65 | 1.03 | 1.00 |
| rs2046210 | 23.29 | 23.29 | 20.47 | 24.26 | 1.11 | 1.06 |
| rs2075555 | 0.40 | 0.40 | 0.27 | 0.72 | 1.02 | 1.00 |
| rs2180341 | 2.12 | 2.12 | 1.53 | 2.28 | 1.06 | 1.00 |
| rs2981582 | 87.61 | 87.61 | 85.90 | 86.64 | 1.23 | 1.18 |
| rs3750817 | 48.21 | 48.21 | 48.74 | 47.42 | 1.19 | 1.13 |
| rs3803662 | 51.69 | 51.68 | 48.87 | 49.49 | 1.16 | 1.19 |
| rs3817198 | 0.03 | 0.03 | 0.01 | 0.02 | 1.01 | 1.02 |
| rs4973768 | 14.70 | 14.70 | 15.25 | 14.56 | 1.11 | 1.04 |
| rs6504950 | 13.66 | 13.66 | 12.04 | 12.79 | 1.10 | 1.10 |
| rs8034191 | 1.35 | 1.36 | 2.17 | 0.68 | 1.00 | 1.09 |
| rs10941679 | 28.11 | 28.11 | 29.12 | 29.86 | 1.09 | 1.18 |
| rs11249433 | 17.70 | 17.70 | 16.89 | 16.21 | 1.12 | 1.01 |
| rs12914385 | 1.08 | 1.09 | 1.71 | 0.60 | 1.01 | 1.07 |
| rs13281615 | 15.44 | 15.44 | 15.68 | 15.10 | 1.06 | 1.12 |
| rs13387042 | 58.18 | 58.18 | 60.59 | 59.12 | 1.17 | 1.15 |
| SUM | 395.16 | 395.16 | 390.86 | 386.83 | n/a | n/a |

Supplementary Table 13. χ^2^ statistics for RA data set. OR Young is the odds ratio computed from cases with age less than the median case age. OR Old is the odds ratio for cases with age greater than median age.

| Rsid | LTPub | LTFit | LogR | LogR+Cov | OR Young | OR Old |
| --- | --- | --- | --- | --- | --- | --- |
| rs2476601 | 78.66 | 78.96 | 70.41 | 69.95 | 1.69 | 1.41 |
| rs13031237 | 14.12 | 14.11 | 13.81 | 13.66 | 1.17 | 1.13 |
| rs10865035 | 10.51 | 10.47 | 11.23 | 10.86 | 1.15 | 1.11 |
| rs7574865 | 15.39 | 15.34 | 15.95 | 15.99 | 1.17 | 1.17 |
| rs3087243 | 14.47 | 14.62 | 11.40 | 11.46 | 1.18 | 1.08 |
| rs2069778 | 11.07 | 11.03 | 11.66 | 11.40 | 1.17 | 1.17 |
| rs6897932 | 6.41 | 6.41 | 6.53 | 6.22 | 1.11 | 1.09 |
| rs9270657 | 161.53 | 161.40 | 151.55 | 150.41 | 1.69 | 1.64 |
| rs6920220 | 23.32 | 23.39 | 21.64 | 21.41 | 1.26 | 1.15 |
| rs182429 | 10.09 | 10.09 | 9.94 | 9.58 | 1.14 | 1.10 |
| rs3093023 | 10.79 | 10.95 | 8.33 | 7.23 | 1.18 | 1.05 |
| rs2736340 | 7.09 | 7.09 | 7.22 | 6.11 | 1.14 | 1.10 |
| rs951005 | 10.42 | 10.43 | 9.80 | 9.76 | 1.16 | 1.18 |
| rs2900180 | 19.13 | 19.13 | 17.76 | 18.76 | 1.16 | 1.16 |
| rs706778 | 8.20 | 8.16 | 8.43 | 8.86 | 1.11 | 1.11 |
| rs4750316 | 12.78 | 12.80 | 12.69 | 12.03 | 1.19 | 1.14 |
| rs2614394 | 55.71 | 54.97 | 44.45 | 43.43 | 7.83 | 12.09 |
| rs1678542 | 9.02 | 8.99 | 8.95 | 9.29 | 1.10 | 1.12 |
| rs4810485 | 14.58 | 14.68 | 12.70 | 12.42 | 1.21 | 1.11 |
| rs11203203 | 11.57 | 11.63 | 10.26 | 10.81 | 1.17 | 1.09 |
| rs3218258 | 6.43 | 6.43 | 6.21 | 6.46 | 1.12 | 1.08 |
| SUM | 511.31 | 511.11 | 470.91 | 466.11 | n/a | n/a |

Supplementary Table 14. χ^2^ statistics for ESKD data set. OR Young is the odds ratio computed from cases with age less than the median case age. OR Old is the odds ratio for cases with age greater than median age.

| Rsid | LT | LogR | LogR+Cov | OR Young | OR Old |
| --- | --- | --- | --- | --- | --- |
| rs73885319 | 188.38 | 137.80 | 134.70 | 3.34 | 1.93 |

Supplementary Table 15. χ^2^ statistics for AMD data set. OR Young is the odds ratio computed from cases with age less than the median case age. OR Old is the odds ratio for cases with age greater than median age.

| Rsid | LT | LogR | LogR+Cov | OR Young | OR Old |
| --- | --- | --- | --- | --- | --- |
| rs1061170 | 91.73 | 80.08 | 59.94 | 2.34 | 2.00 |
| rs10490924 | 93.87 | 79.29 | 50.39 | 2.50 | 2.18 |
| SUM | 185.60 | 159.38 | 110.33 | n/a | n/a |

1. Lipton RB, Liao Y, Cao G, Cooper RS, McGee D (1993) Determinants of incident non-insulin-dependent diabetes mellitus among blacks and whites in a national sample. The NHANES I Epidemiologic Follow-up Study. Am J Epidemiol 138: 826-839.

2. Narayan KM, Boyle JP, Thompson TJ, Gregg EW, Williamson DF (2007) Effect of BMI on lifetime risk for diabetes in the U.S. Diabetes Care 30: 1562-1566.

3. Godtfredsen NS, Prescott E, Osler M (2005) Effect of smoking reduction on lung cancer risk. JAMA 294: 1505-1510.

4. Stolt P, Bengtsson C, Nordmark B, Lindblad S, Lundberg I, et al. (2003) Quantification of the influence of cigarette smoking on rheumatoid arthritis: results from a population based case-control study, using incident cases. Ann Rheum Dis 62: 835-841.

5. Friedman DS, O'Colmain BJ, Munoz B, Tomany SC, McCarty C, et al. (2004) Prevalence of age-related macular degeneration in the United States. Arch Ophthalmol 122: 564-572.

6. Kiberd BA, Clase CM (2002) Cumulative risk for developing end-stage renal disease in the US population. J Am Soc Nephrol 13: 1635-1644.

7. Chatterjee N, Carroll RJ (2005) Semiparametric maximum-likelihood estimation exploiting gene-environment independence in case-control studies. Biometrika 92: 19.

8. Cornelis MC, Tchetgen EJ, Liang L, Qi L, Chatterjee N, et al. (2012) Gene-environment interactions in genome-wide association studies: a comparative study of tests applied to empirical studies of type 2 diabetes. Am J Epidemiol 175: 191-202.

9. Tchetgen Tchetgen EJ, Kraft P (2011) On the robustness of tests of genetic associations incorporating gene-environment interaction when the environmental exposure is misspecified. Epidemiology 22: 257-261.

10. Wray NR, Yang J, Goddard ME, Visscher PM (2010) The genetic interpretation of area under the ROC curve in genomic profiling. PLoS Genet 6: e1000864.

11. Sasieni PD (1997) From genotypes to genes: doubling the sample size. Biometrics 53: 1253-1261.

12. Voight BF, Scott LJ, Steinthorsdottir V, Morris AP, Dina C, et al. (2010) Twelve type 2 diabetes susceptibility loci identified through large-scale association analysis. Nat Genet 42: 579-589.

13. Kooner JS, Saleheen D, Sim X, Sehmi J, Zhang W, et al. (2011) Genome-wide association study in individuals of South Asian ancestry identifies six new type 2 diabetes susceptibility loci. Nat Genet 43: 984-989.

14. Yamauchi T, Hara K, Maeda S, Yasuda K, Takahashi A, et al. (2010) A genome-wide association study in the Japanese population identifies susceptibility loci for type 2 diabetes at UBE2E2 and C2CD4A-C2CD4B. Nat Genet 42: 864-868.

15. Dupuis J, Langenberg C, Prokopenko I, Saxena R, Soranzo N, et al. (2010) New genetic loci implicated in fasting glucose homeostasis and their impact on type 2 diabetes risk. Nat Genet 42: 105-116.

16. Sandhu MS, Weedon MN, Fawcett KA, Wasson J, Debenham SL, et al. (2007) Common variants in WFS1 confer risk of type 2 diabetes. Nat Genet 39: 951-953.

17. Gudmundsson J, Sulem P, Steinthorsdottir V, Bergthorsson JT, Thorleifsson G, et al. (2007) Two variants on chromosome 17 confer prostate cancer risk, and the one in TCF2 protects against type 2 diabetes. Nat Genet 39: 977-983.

18. Zeggini E, Scott LJ, Saxena R, Voight BF, Marchini JL, et al. (2008) Meta-analysis of genome-wide association data and large-scale replication identifies additional susceptibility loci for type 2 diabetes. Nat Genet 40: 638-645.

19. Unoki H, Takahashi A, Kawaguchi T, Hara K, Horikoshi M, et al. (2008) SNPs in KCNQ1 are associated with susceptibility to type 2 diabetes in East Asian and European populations. Nat Genet 40: 1098-1102.

20. Lyssenko V, Nagorny CL, Erdos MR, Wierup N, Jonsson A, et al. (2009) Common variant in MTNR1B associated with increased risk of type 2 diabetes and impaired early insulin secretion. Nat Genet 41: 82-88.

21. (2007) Genome-wide association study of 14,000 cases of seven common diseases and 3,000 shared controls. Nature 447: 661-678.

22. Okamoto K, Iwasaki N, Nishimura C, Doi K, Noiri E, et al. (2010) Identification of KCNJ15 as a susceptibility gene in Asian patients with type 2 diabetes mellitus. Am J Hum Genet 86: 54-64.

23. Salonen JT, Uimari P, Aalto JM, Pirskanen M, Kaikkonen J, et al. (2007) Type 2 diabetes whole-genome association study in four populations: the DiaGen consortium. Am J Hum Genet 81: 338-345.

24. Kote-Jarai Z, Olama AA, Giles GG, Severi G, Schleutker J, et al. (2011) Seven prostate cancer susceptibility loci identified by a multi-stage genome-wide association study. Nat Genet 43: 785-791.

25. Haiman CA, Chen GK, Blot WJ, Strom SS, Berndt SI, et al. (2011) Genome-wide association study of prostate cancer in men of African ancestry identifies a susceptibility locus at 17q21. Nat Genet 43: 570-573.

26. Takata R, Akamatsu S, Kubo M, Takahashi A, Hosono N, et al. (2010) Genome-wide association study identifies five new susceptibility loci for prostate cancer in the Japanese population. Nat Genet 42: 751-754.

27. Yeager M, Chatterjee N, Ciampa J, Jacobs KB, Gonzalez-Bosquet J, et al. (2009) Identification of a new prostate cancer susceptibility locus on chromosome 8q24. Nat Genet 41: 1055-1057.

28. Gudmundsson J, Sulem P, Gudbjartsson DF, Blondal T, Gylfason A, et al. (2009) Genome-wide association and replication studies identify four variants associated with prostate cancer susceptibility. Nat Genet 41: 1122-1126.

29. Eeles RA, Kote-Jarai Z, Al Olama AA, Giles GG, Guy M, et al. (2009) Identification of seven new prostate cancer susceptibility loci through a genome-wide association study. Nat Genet 41: 1116-1121.

30. Al Olama AA, Kote-Jarai Z, Giles GG, Guy M, Morrison J, et al. (2009) Multiple loci on 8q24 associated with prostate cancer susceptibility. Nat Genet 41: 1058-1060.

31. Sun J, Zheng SL, Wiklund F, Isaacs SD, Purcell LD, et al. (2008) Evidence for two independent prostate cancer risk-associated loci in the HNF1B gene at 17q12. Nat Genet 40: 1153-1155.

32. Eeles RA, Kote-Jarai Z, Giles GG, Olama AA, Guy M, et al. (2008) Multiple newly identified loci associated with prostate cancer susceptibility. Nat Genet 40: 316-321.

33. Thomas G, Jacobs KB, Yeager M, Kraft P, Wacholder S, et al. (2008) Multiple loci identified in a genome-wide association study of prostate cancer. Nat Genet 40: 310-315.

34. Witte JS (2007) Multiple prostate cancer risk variants on 8q24. Nat Genet 39: 579-580.

35. Yeager M, Orr N, Hayes RB, Jacobs KB, Kraft P, et al. (2007) Genome-wide association study of prostate cancer identifies a second risk locus at 8q24. Nat Genet 39: 645-649.

36. Amundadottir LT, Sulem P, Gudmundsson J, Helgason A, Baker A, et al. (2006) A common variant associated with prostate cancer in European and African populations. Nat Genet 38: 652-658.

37. Haiman CA, Patterson N, Freedman ML, Myers SR, Pike MC, et al. (2007) Multiple regions within 8q24 independently affect risk for prostate cancer. Nat Genet 39: 638-644.

38. Wen W, Cho YS, Zheng W, Dorajoo R, Kato N, et al. (2012) Meta-analysis identifies common variants associated with body mass index in east Asians. Nat Genet 44: 307-311.

39. Bonnefond A, Clement N, Fawcett K, Yengo L, Vaillant E, et al. (2012) Rare MTNR1B variants impairing melatonin receptor 1B function contribute to type 2 diabetes. Nat Genet 44: 297-301.

40. Akamatsu S, Takata R, Haiman CA, Takahashi A, Inoue T, et al. (2012) Common variants at 11q12, 10q26 and 3p11.2 are associated with prostate cancer susceptibility in Japanese. Nat Genet 44: 426-429, S421.

41. Qin X, Hauser ER, Schmidt S (2010) Ordered subset analysis for case-control studies. Genet Epidemiol 34: 407-417.

42. Monsees GM, Tamimi RM, Kraft P (2009) Genome-wide association scans for secondary traits using case-control samples. Genet Epidemiol 33: 717-728.

43. Rose S, van der Laan M (2008) Simple Optimal Weighting of Cases and Controls in Case-Control Studies. The International Journal of Biostatistics 4.

44. Murcray CE, Lewinger JP, Conti DV, Thomas DC, Gauderman WJ (2011) Sample size requirements to detect gene-environment interactions in genome-wide association studies. Genet Epidemiol 35: 201-210.

45. Price AL, Patterson NJ, Plenge RM, Weinblatt ME, Shadick NA, et al. (2006) Principal components analysis corrects for stratification in genome-wide association studies. Nat Genet 38: 904-909.

46. Zaitlen N, Pasaniuc B, Patterson N, Pollack S, Voight B, et al. (2012) Analysis of case-control association studies with known risk variants. Bioinformatics 28: 1729-1737.

47. Lindstrom S, Schumacher F, Siddiq A, Travis RC, Campa D, et al. (2011) Characterizing Associations and SNP-Environment Interactions for GWAS-Identified Prostate Cancer Risk Markers-Results from BPC3. PLoS One 6: e17142.
